# Synthesis and properties of sulfur-functionalized triarylmethylium, acridinium and triangulenium dyes

**DOI:** 10.3762/bjoc.15.210

**Published:** 2019-09-09

**Authors:** Marco Santella, Eduardo Della Pia, Jakob Kryger Sørensen, Bo W Laursen

**Affiliations:** 1 Nano-Science Center and Department of Chemistry, University of Copenhagen, Universitetsparken 5, 2100 Copenhagen Ø, Denmark

**Keywords:** acridinium dyes, aromatic nucleophilic substitution, fluorescent dyes, sulfur-functionalized dyes, triangulenium dyes, triarylmethylium

## Abstract

Triangulenium dyes functionalized with one, two or three ethylthiol functionalities were synthesized and their optical properties were studied. The sulfur functionalities were introduced by aromatic nucleophilic substitution of methoxy groups in triarylmethylium cations with ethanethiol followed by partial or full ring closure of the *ortho* positions with nitrogen or oxygen bridges leading to sulfur-functionalized acridinium, xanthenium or triangulenium dyes. For all the dye classes the sulfur functionalities are found to lead to intensely absorbing dyes in the visible range (470 to 515 nm), quite similar to known analogous dye systems with dialkylamino donor groups in place of the ethylthiol substituents. For the triangulenium derivatives significant fluorescence was observed (Φ_f_ = 0.1 to Φ_f_ = 0.3).

## Introduction

The design, synthesis and studies of organic fluorescent dyes have witnessed a revival in recent years, in particular due to their applications in imaging and biomedical assays and analytical techniques [1–5]. The desire to detect minute amounts of dye, ideally single molecules [6–7], in complex biological environments with high levels of autofluorescence, constantly challenges chemists to develop new dyes with improved or special properties. In the design of simple dyes parameters such as molar absorption coefficients (ε), absorption/emission wavelengths [8–9], fluorescence quantum yields (Φ_fl_) [10–11], and fluorescence lifetime (τ_fl_) [12–13] are key photophysical properties to consider and optimize for any given application.

**Figure 1:**
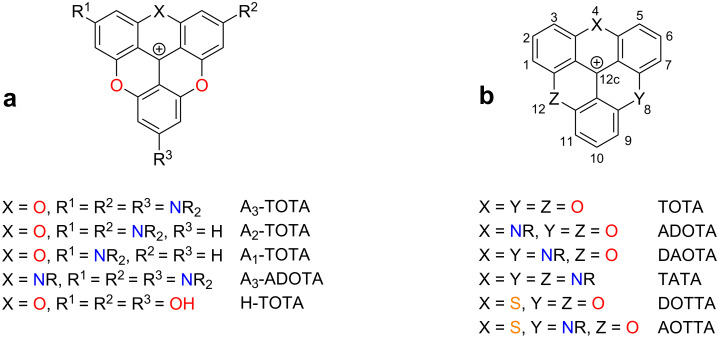
Structures of some representative triangulenium dyes. a) Rhodamine/fluorescine-like derivatives with donor groups in *para*-positions (2, 6, and 10) to the formal cation center (12c). b) Derivatives without donor groups.

We have for quite some time been interested in the synthesis, properties and applications of dyes from the triangulenium family (Figure 1) [14–15]. The triangulenium dyes can be divided into two main categories: 1) triangulenium dyes with donor substituents at the corners of the triangulenium ring system (position 2, 6 and 10, Figure 1a) [16–18], and 2) triangulenium dyes without such groups (Figure 1b) [19–21]. Dyes in the first category have intense absorption (ε ≈ 50,000–130,000 M^−1^·cm^−1^), high fluorescence quantum yields (Φ_fl_ > 50%) and fluorescence lifetimes of 3–4 ns. All properties that agree well with their structural resemblance to rhodamines and fluoresceines, and triangulenium dyes such as A_3_-TOTA^+^ and H-TOTA^+^ (Figure 1a) can be viewed as extended symmetric versions of these prominent dyes [16,22]. The second class of triangulenium dyes, without appended donor groups, are characterized by much less intense transitions (ε ≈ 5,000–20,000 M^−1^·cm^−1^), which for some derivatives leads to unusually long fluorescence lifetimes (τ_fl_ ≈ 20 ns) [23–24]. This long fluorescence lifetime has been a key point of interest since it enables time-gated detection for suppression of autofluorescence [25–26] and provides attractive advantages in fluorescence polarization assays [13,27–28].

**Figure 2:**
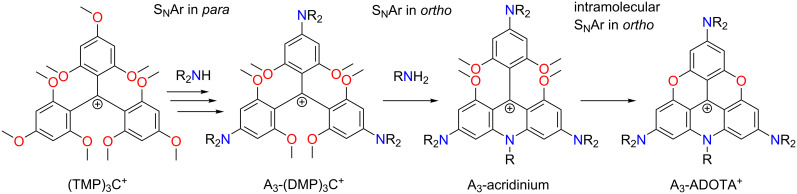
Examples of various types of S_N_Ar reactions typical in triangulenium synthesis, exemplified with the synthesis of A_3_-ADOTA^+^: step 1, replacement of *p*-methoxy groups with dialkylamines. Step 2, replacement of *o-*methoxy groups with a primary amine followed by intramolecular S_N_Ar reaction. Step 3, intramolecular S_N_Ar replacement of methoxy groups by hydroxy groups formed under ether-cleavage conditions.

A common characteristic feature of triangulenium dye synthesis is the use of methoxy-substituted triarylmethylium salts as simple precursors allowing both introduction of dialkylamino donor groups and formation of the heterocyclic triangulenium ring systems. These characteristic types of aromatic nucleophilic substitution (S_N_Ar) reactions are exemplified by the synthesis of A_3_-ADOTA^+^ (Figure 2) [17]. Starting from the readily available tris(2,4,6-trimethoxyphenyl)methylium salt (TMP)_3_C^+^ [18,29], stepwise replacement of the *para-*methoxy groups by dialkylamines provides access to a wide variety of symmetric and asymmetric triarylmethylium dyes [18,30–31]. Replacement of two *o-*methoxy groups by one primary amine gives acridinium-type ring systems (Figure 2, step 2) and is a key reaction for the formation of the unsubstituted triangulenium dyes shown in Figure 1b [19–20]. Finally, formation of oxygen bridges in the triangulenium system (Figure 2, step 3) involves demethylation conditions and intramolecular S_N_Ar replacement of *ortho-*methoxy groups [18,32].

The S_N_Ar approach to the synthesis of triangulenium dyes [14,18–19] has been extremely successful and expanded the family greatly from the single derivative (TOTA^+^, Figure 1b) first synthesized by Martin and Smith in 1964 [32], and also includes the family of helicenium dyes [33–35]. However, the introduction of groups other than nitrogen and oxygen has so far not been performed by the S_N_Ar approach. Thus in the preparation of the sulfur-bridged triangulenium ions DOTTA^+^ and AOTTA^+^ (Figure 1b) Lacour and co-workers reported unsuccessful attempts of S_N_Ar reactions with sulfur nucleophiles in *ortho-*position of (TMP)_3_C^+^ and had to assemble the thioxanthenium part of the triangulenium ring system independently by other means [36]. Similarly, we had to use a stepwise buildup of the triangulenium systems to introduce saturated [37] and unsaturated [38] carbon bridges.

Here we report for the first time the introduction of sulfur functionalities into triangulenium dyes by S_N_Ar reaction with ethylthiol nucleophiles in the *para-*positions accessing several new families of xanthenium, acridinium and triangulenium dyes with thioether donor groups.

## Results and Discussion

**Scheme 1:**
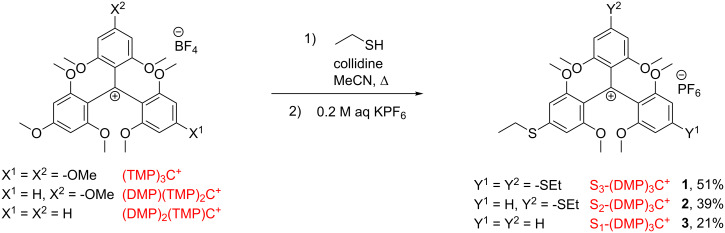
Synthesis of three novel S_X_-(DMP)_3_C^+^ PF_6_^−^ ethylsulfanyl-substituted triarylmethylium salts.

Firstly, a series of triarylmethylium salts with variable number of *para-*methoxy substituents was synthesized. The easily achievable cations (TMP)_3_C^+^, (DMP)(TMP)_2_C^+^ and (DMP)_2_(TMP)C^+^ (Scheme 1) were prepared by their respective literature procedures [18,31]. To investigate the reactivity of these carbenium systems in S_N_Ar reactions with sulfur-based nucleophiles, simple alkylthiols were chosen, with the ethyl and *tert*-butyl thiols being the primary choice. S_N_Ar reactions with the two thiols were tested under identical reaction conditions (Scheme 1).

These conditions consisted of heating the reaction components in refluxing acetonitrile in the presence of collidine as base. For all three carbenium salts examinations showed that only ethanethiol lead to substitution. The progress of the reaction was conveniently followed by MALDI–TOF mass spectrometry. In case of the reactions with *tert*-butylthiolate, neither detection of the target molecule nor any of the intermediates were observed. This lack of reactivity is likely explained by the *tert*-butylthiolate nucleophile being too bulky to undergo reaction. In the successful reactions, which had occurred with ethanethiol, a high selectivity was observed for *para*-substitutions, giving S_x_-(DMP)_3_C^+^ **1**, **2**, and **3** in reasonable yields of 20–50% after column chromatography purification. It is important to note that the gradual introduction of thioethers into the carbenium systems did not significantly influence the overall reactivity of the system towards subsequent nucleophilic aromatic substitution. When the reaction was followed by MALDI–TOFMS spectrometry it was thus possible to observe simultaneously the presence of the target compound and all of the intermediates involved in the reaction. This behavior is contrary to the reaction pattern observed when using dialkylamines as nucleophiles, where the strong electron-donating effect of the introduced amines stabilize the carbenium ion products and thus significantly reduces the reactivity of the remaining methoxy groups for further substitutions [18,39]. This observation is in agreement with the much stronger cation stabilization of the dialkylamino group compared to the methoxy group. The ability of the alkylthio group to stabilize carbenium ions, given by the Hammet σ_p_^+^ value [40], on the other hand is quite similar to the methoxy group or even a little lower [41], and does thus not slow down the multistep S_N_Ar reactions.

**Scheme 2:**
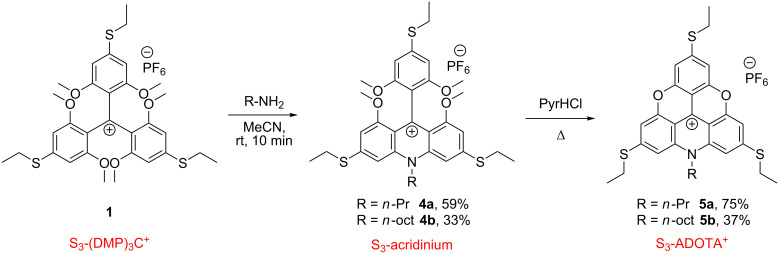
Synthetic route for the synthesis of S_3_-ADOTA^+^.

The new *ortho*-methoxytriarylcarbenium ions with one, two and three *para*-SEt groups (**1**–**3**) are potential precursors for a wide variety of new triangulenium, xanthenium, and acridinium dyes. To elucidate some of these possibilities we first investigated transformations of the symmetric derivative **1**. Treatment with primary alkylamines, *n*-propylamine and *n*-octylamine, yielded exclusively the acridinium products **4a**,**b** (Scheme 2). This *ortho* S_N_Ar transformation is similar to what is reported for the (DMP)_3_C^+^ system [19–20,42] lacking *para*-substituents and for the *para*-amino-substituted analogue [17] (step 2 in Figure 1). It was found that the reactivity in S_N_Ar reactions of **1** with primary amines was high and the acridinium compounds **4a**,**b** were obtained in few minutes after the addition of 2 equiv of the corresponding primary amine at room temperature. Further ring closure to two oxygen bridges in acridinium compounds **4a** and **4b** to the corresponding triangulenium dyes S_3_-ADOTA^+^ (**5a**,**b**) was achieved by heating in molten pyridine hydrochloride (Scheme 2).

It is noteworthy that the ethylthio ether linkages remained intact upon treatment with molten PyrHCl, which was found to result in complicated mixtures of dealkylated byproducts when these conditions were applied on dialkylamino-substituted carbenium systems [18].

**Scheme 3:**
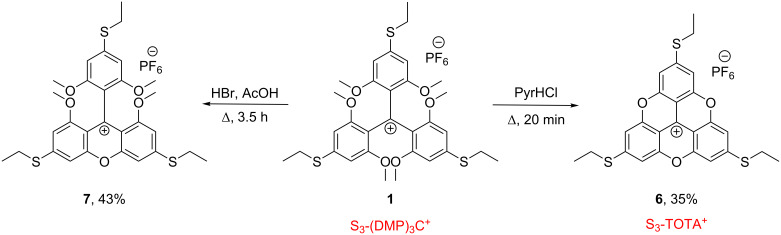
Synthesis of S_3_-TOTA^+^ PF_6_^−^ (**6**) and the mono ring closed xanthenium **7**.

The direct ring closure of **1** in PyrHCl yielded in a similar manner the sulfur-functionalized trioxatriangulenium system S_3_-TOTA^+^ (**6**) as shown in Scheme 3. Mono ring closure was achieved under milder ether cleaving conditions with aqueous HBr in acetic acid, leading to the ethylthio-substituted xanthenium system **7** in 43% yield (Scheme 3).

**Scheme 4:**
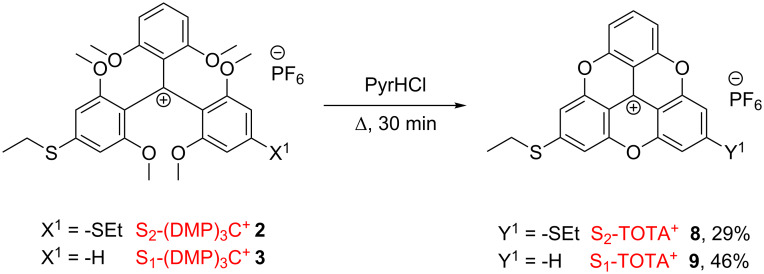
Synthesis of S_2_-TOTA^+^ PF_6_^−^ (**8**) and S_1_-TOTA^+^ PF_6_^−^ (**9**).

By applying similar molten pyridine hydrochloride conditions to the mono- and disubstituted thioether carbenium salts (**2** and **3**), it was possible to isolate the derivatives S_2_-TOTA^+^ (**8**) and S_1_-TOTA^+^ (**9**), respectively as their hexafluorophosphate salts (Scheme 4). The two S_x_-TOTA^+^ derivatives were obtained with good yield after purification by column chromatography and subsequent recrystallization.

To conclude, the successful introduction of -SEt groups by the S_N_Ar approach, and subsequent nitrogen and oxygen ring-closure reactions provides access to several new families of carbenium dyes, all with the unusual -SR donor group: thus **1**–**3** represent new triarylmethylium dyes, **4a** and **4b** sulfur analogues of aminoacridinium dyes (acridine orange-like structures), **7** a fluorescein-like xanthenium dye, **5a** and **5b** are sulfur-substituted ADOTA^+^ dyes, and finally the three sulfur-substituted TOTA^+^ dyes **6**, **8** and **9**.

Now the relevant questions are: how do the -SR donor groups influence transition energies and intensities? And how do they affect fluorescence quantum yields in these new dye systems? To the extent possible we will compare the new sulfur-functionalized dyes to known analogues with -OR or -NR_2_ donor groups in the same positions.

**Figure 3:**
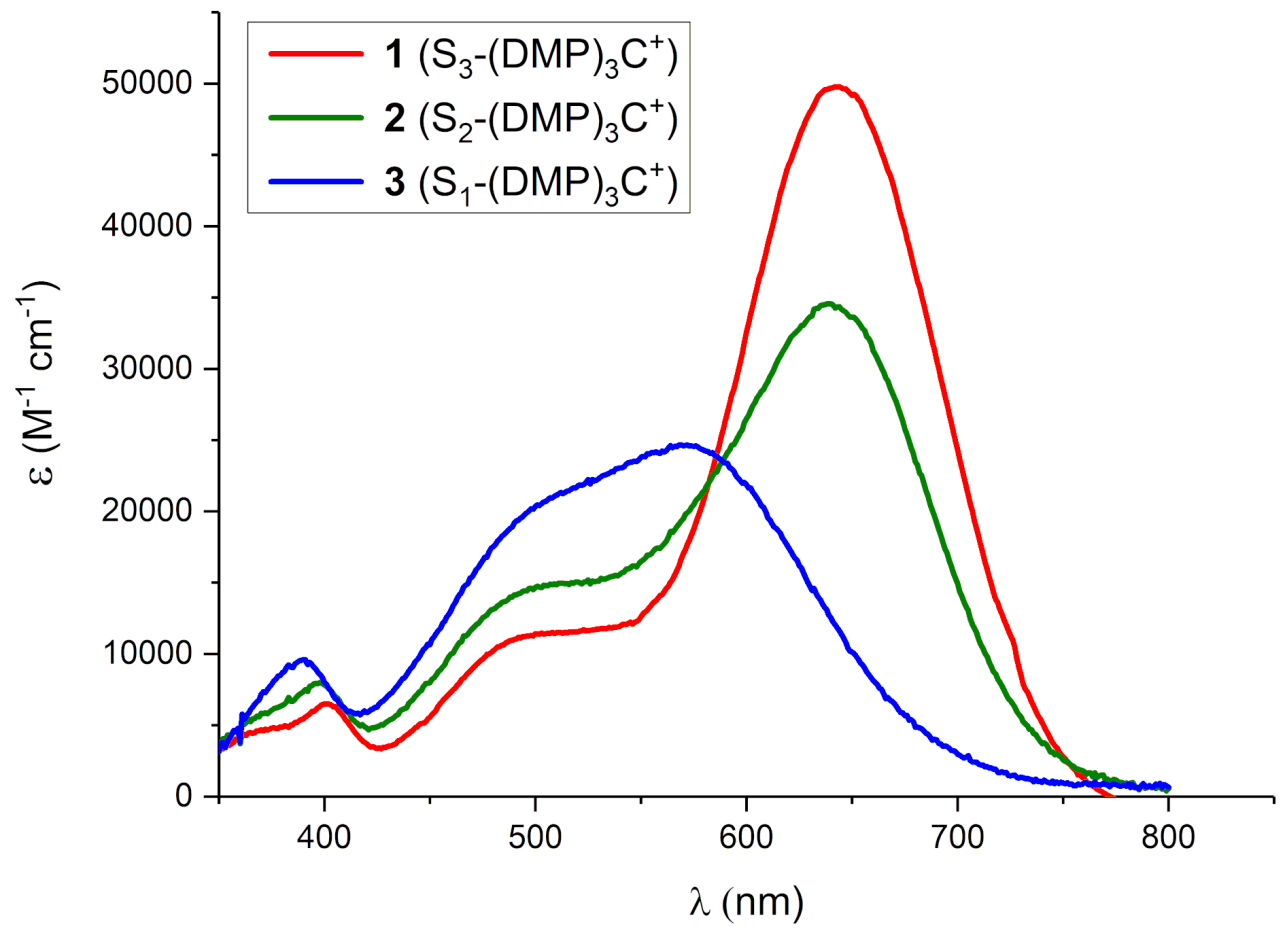
UV–vis spectra in MeCN: S_3_-(DMP)_3_C^+^ (**1**, red), S_2_-(DMP)_3_C^+^ (**2**, green), and S_1_-(DMP)_3_C^+^ (**3**, blue).

The sulfur-substituted triarylmethylium dyes **1, 2** and **3** display broad absorption bands in the 500–700 nm region (Figure 3), that in shape and relative transition energy are quite similar to the analogues with similar numbers of *para*-methoxy or diethylamino groups [31], as shown by comparison of maximum absorption wavelength (λ_max,abs_) and molar absorptivity (ε) in Table 1. It is noticed that the -SEt donor group in these *ortho*-hexamethoxytriarylmethylium dyes provides transition energies and intensities very similar to those of commonly used dialkylamino-donor groups, but significantly red-shifted relative to the methoxy-substituted analogues.

**Table 1:** Summary of absorption data of triarylmethylium ions in MeCN.

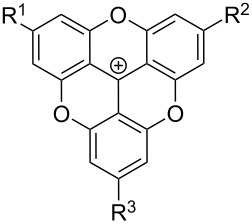			

	λ_max.abs._ (ε, M^−1^·cm^−1^)		
	
donor groups R^1^, R^2^, R^3^	-SEt	-OMe^a^	-NEt_2_^a^

one donor R^2^ = R^3^ = H	576 nm (24600)	491 nm (14100)	457 nm (16900)
two donors R^3^ = H	639 nm (34600)	580 nm (18400)	637 nm (40400)
three donors	642 nm (49800)	577 nm (23400)	634 nm (49400)

^a^Data from [43–44].

**Figure 4:**
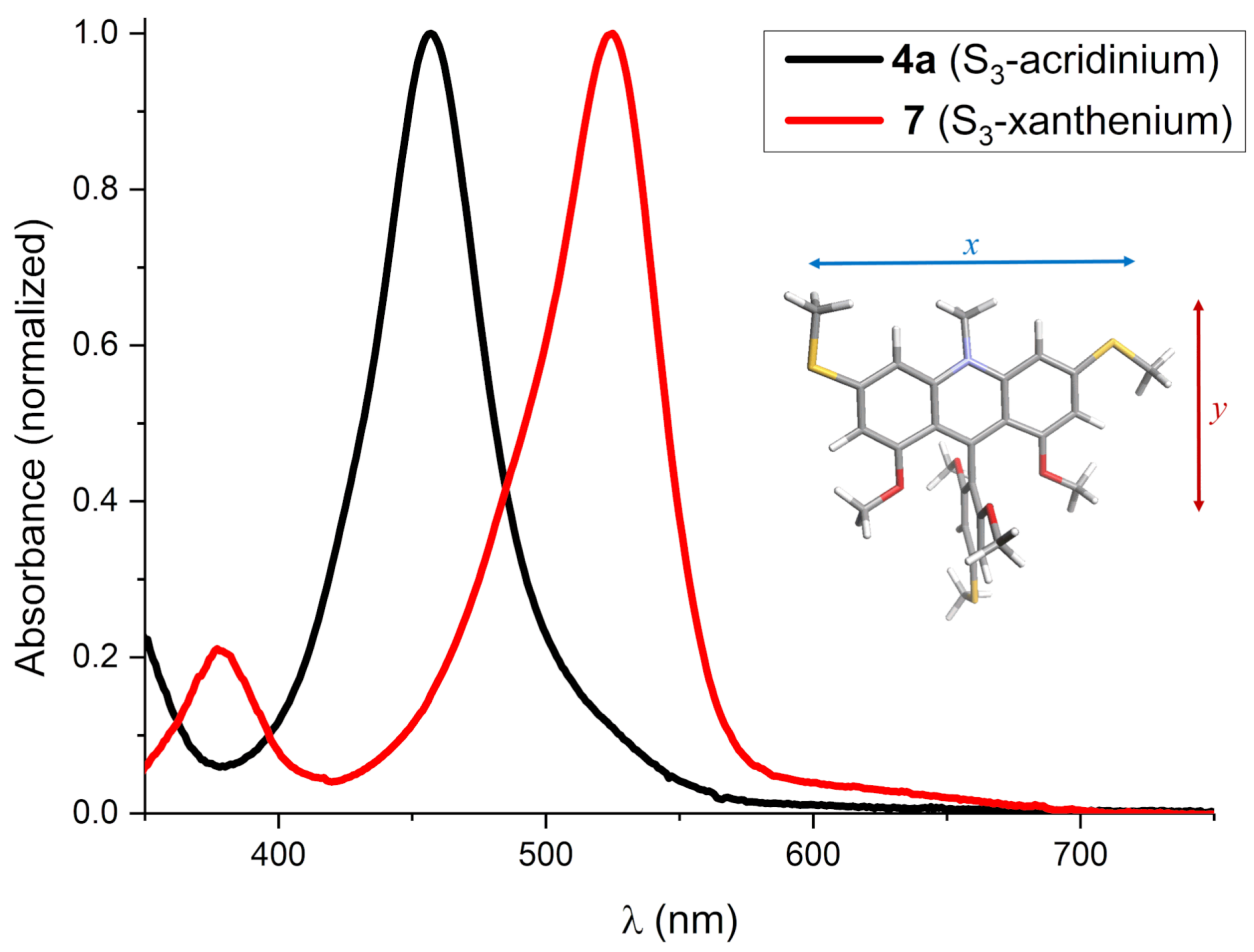
UV–vis spectra in MeCN: S_3_-acridinium (**4a**, black) and S_3_-xanthenium (**7**, red). Inset: The 3D structure of **4a** with indication of the principle axes of the electronic transitions.

Absorption spectra of the partially ring-closed acridinium and xanthenium compounds, with three *para*-SEt groups, **4a** and **7**, respectively, are shown in Figure 4. For these compounds the spectra are dominated by strong transitions assigned to the 3,6-diethylthio-acridinium and -xanthenium ring systems peaking at 457 nm (ε = 47000 M^−1^·cm^−1^) and at 520 nm (ε = 60000 M^−1^·cm^−1^), respectively (see Supporting Information File 1, Table S1 for additional data in more solvents). The energy and intensity of these transitions are quite similar to those found in dialkylamino analogues, that are 3,6-diaminoacridines and rhodamines [43–44]. The weak tails on the red side of these bands are tentatively assigned to internal charge-transfer transitions from the perpendicularly [19,42] arranged ethylthio(dimethoxy)phenyl group to the xanthenium/acridinium systems polarized along the *y*-axes (Figure 4, inset). This bichromophoric behavior has been studied in detail for the dialkylamino-substituted xanthenium/rhodamine system [45–46], and is also the likely reason for these compounds being non-fluorescent.

**Figure 5:**
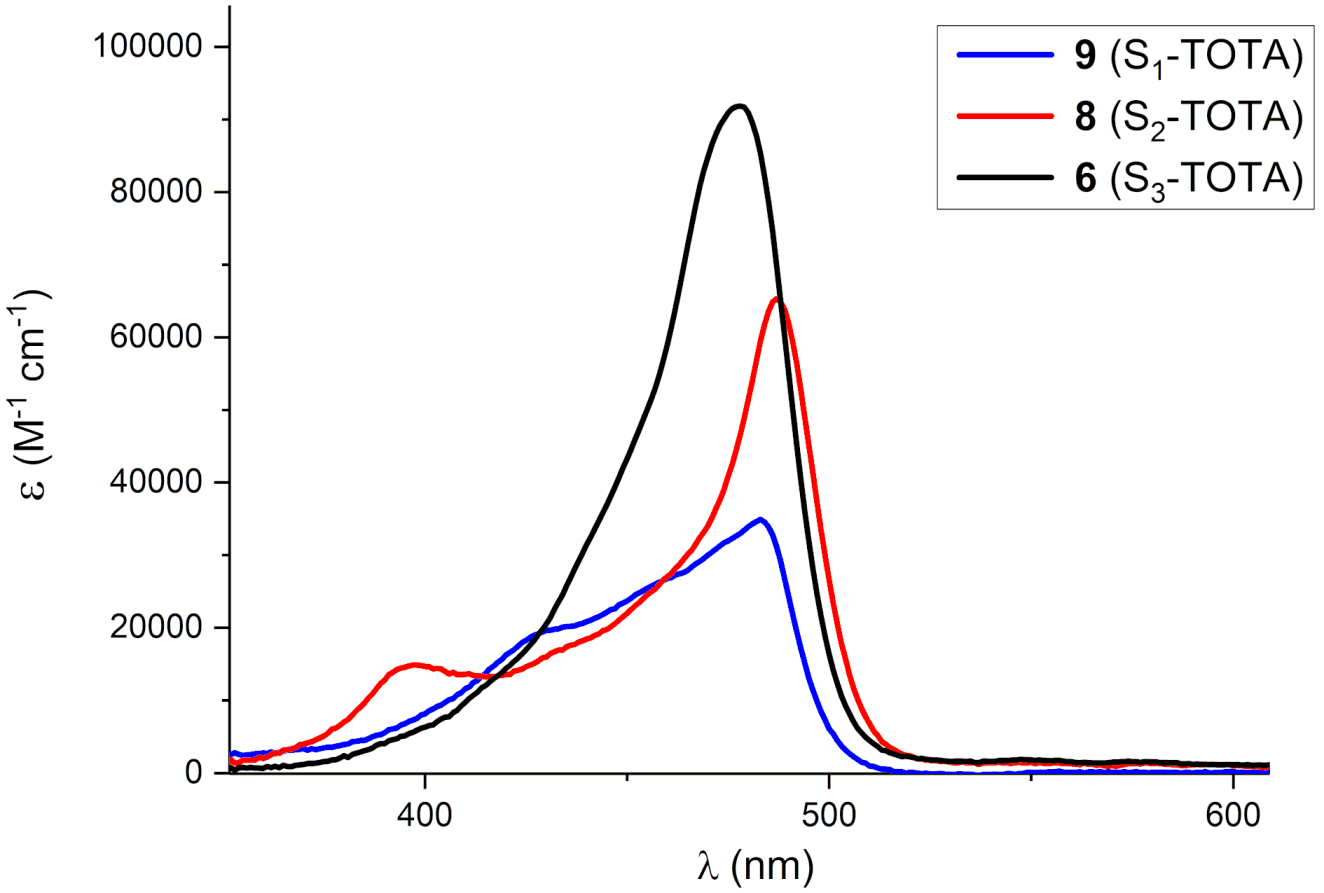
UV–vis spectra in CH_2_Cl_2_: S_1_-TOTA^+^ (**9**, blue line), S_2_-TOTA^+^ (**8**, red line), and S_3_-TOTA^+^ (**6**, black line).

The three sulfur-substituted trioxatriangulenium dyes **6**, **8**, and **9** all display a first absorption band around 480 nm (Figure 5), with increasing intensity as the number of -SEt groups on the TOTA^+^ core increases. This behavior resembles the trend observed for the analogue series of amino-substituted TOTA’s (Table 2) [18,31]. In the two low-symmetry derivatives **8** and **9** transitions to the S_2_ excited states are observed at around 400 nm, while the *D*_3_*_h_* symmetric S_3_-TOTA^+^ shows only one, though broad, absorption band corresponding to merging of the S_1_ and S_2_ transitions into one, arising from the degenerated HOMO in the symmetric dye. The influence of solvent and counter ions on such degenerate states have been studied in detail for the A_3_-TOTA system [22,47] and related triarylmethylium dyes such as crystal violet [48–49].

**Table 2:** Summary of absorption data of substituted TOTA dyes in CH_2_Cl_2_.

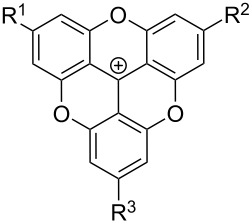		

	λ_max,abs_ (ε, M^−1^·cm^−1^)	
	
Donor groups R^1^, R^2^, R^3^	-SEt	-NEt_2_

one donor R^2^ = R^3^ = H	S_1_-TOTA^+^ (**9**) 483 nm (35000)	A_1_-TOTA^+a^ 507 nm (41700)
two donors R^3^ = H	S_2_-TOTA^+^ (**8**) 487 nm (65200)	A_2_-TOTA^+a^ 513 nm (59700)
three donors	S_3_-TOTA^+^ (**6**) 478 nm (91900)	A_3_-TOTA^+b^ 471 nm (132900)

^a^Data from [31]; ^b^data from [18].

**Figure 6:**
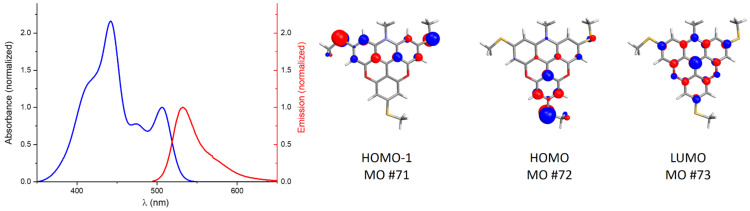
UV–vis absorption and fluorescence spectra (λ_ex_ = 485 nm) of **5a** in CH_2_Cl_2_ solution. Calculated molecular orbital contour plots (semi-empirical method AM1).

When three -SEt groups are placed on the asymmetric azadioxatriangulenium core, as in S_3_-ADOTA^+^ (**5a**), the presence of two electronic transitions becomes very clear, with two well-resolved peaks in the absorption spectrum (Figure 6). The transition at 442 nm is assigned to the S_0_ → S_2_ transition and nearly coincides with the main transition observed in the S_3_-acridinium (**4a**) precursor before ring closure (Figure 4), indicating that this, the most intense transition belongs to the same chromophore, now part of the triangulenium ring system. The S_0_ → S_1_ transition in **5a** is found at 507 nm, where the open form only had a very weak shoulder in its absorption spectrum (Figure 4). The ring closure of **4a** into the fully planar triangulenium system **5a** leads to a significant increase in the orbital overlap and thus also in the intensity of the S_0_ → S_1_ transition. This assignment is supported by calculations of the orbitals involved in the first two electronic transitions (Figure 6), which confirm their localization in different parts of the ADOTA^+^ system. The much more allowed S_0_ → S_1_ transition is also in agreement with the observation that **5a** (and **5b**) display intense fluorescence (Figure 6).

**Figure 7:**
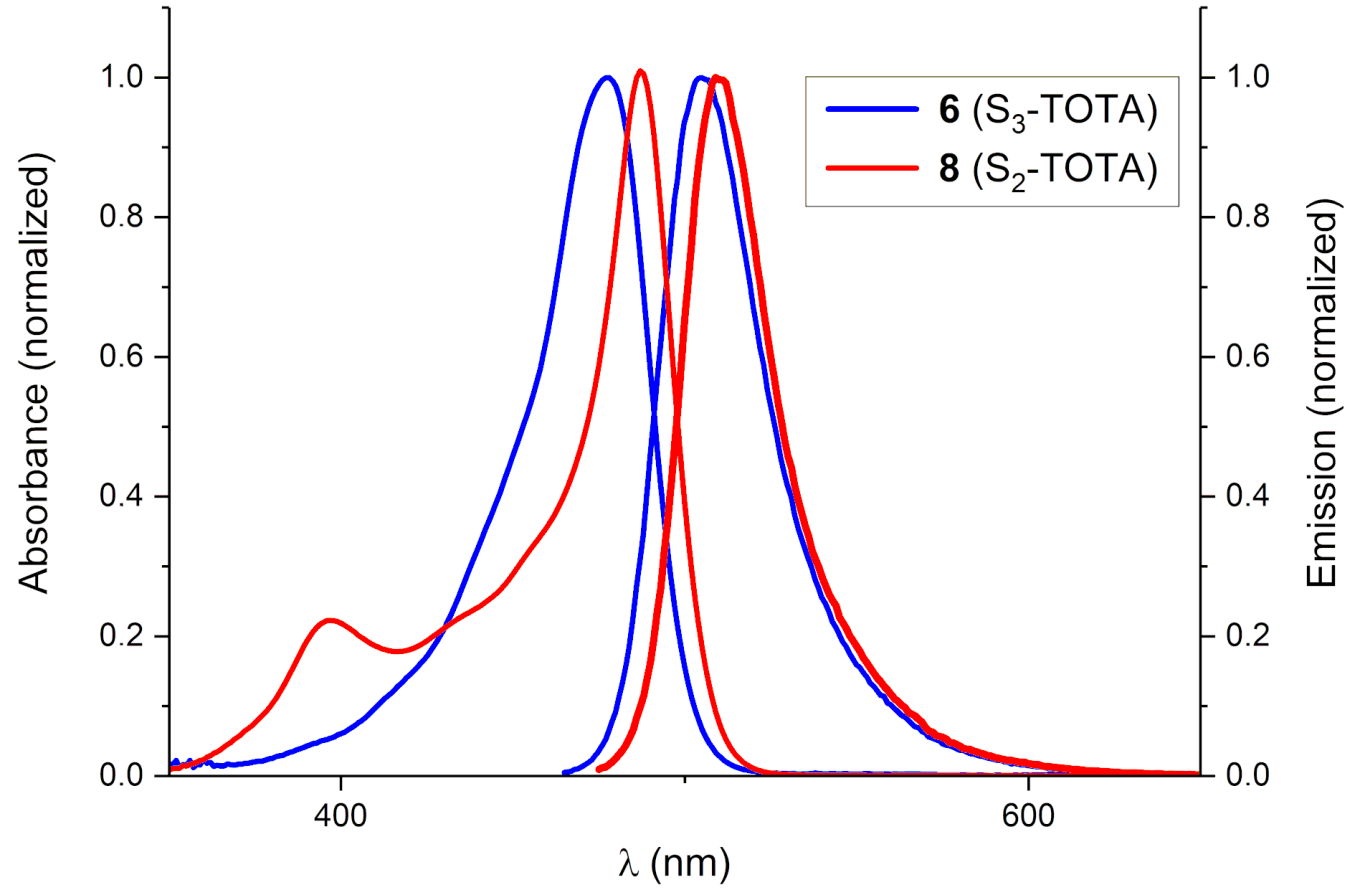
Normalized absorption and fluorescence spectra of **6** (S_3_-TOTA^+^), λ_ex_ = 460 nm, and **8** (S_2_-TOTA^+^), λ_ex_ = 470 nm, in CH_2_Cl_2_ solution.

Table 3 summarizes the spectral and photophysical properties on the triangulenium dyes showing any applicable fluorescence. Beside S_3_-ADOTA^+^ (**5a**) that are the double and triple -SEt-substituted TOTAs **6** and **8**, for which the fluorescence spectra are shown in Figure 7, with fluorescence quantum yields of 16% and 12%, respectively. From the measured fluorescence lifetimes and quantum yields (Table 3) it is possible to calculate the radiative lifetimes (τ_0_), which are found to be in qualitative agreement with the molar absorption coefficients (ε) for the corresponding transitions, as expected from the Strickler–Berg relation [50].

**Table 3:** Summary of optical properties of the fluorescent derivatives.

Compound	Solvent	λ_max,abs_ (nm)	ε (M^−1^·cm^−1^)	λ_max,fl_ (nm)	Φ_f_^a^	τ (ns)	τ_0_^b^ (ns)

**5a** (S_3_-ADOTA)	CH_2_Cl_2_	442 507	76700 35400	532	0.28	3.9	13.9
**6** (S_3_-TOTA)	CH_2_Cl_2_	478	91850	505	0.16	0.7	4.4
**8** (S_2_-TOTA)	CH_2_Cl_2_	487	65200	509	0.12	0.7	5.8

^a^Measured relative to fluorescein in 0.1 M aqueous NaOH (Φ = 0.96); ^b^radiative lifetime τ_0_ = Φ_f_/τ.

While the spectral properties of the new -SEt-substituted dyes are surprisingly similar to the -NEt_2_-substituted analogues across the various dye families they are obviously less bright fluorophores. Thus, the dialkylamino-substituted analogue of **5a** (A_3_-ADOTA^+^) has a reported quantum yield as high as 64% in acetonitrile [17], on par with A_3_-TOTA^+^ and A_2_-TOTA^+^ which display quantum yields from 50–100% depending on the solvent [31]. A similar reduction in fluorescence efficiency was observed by Kotaskova et al. for fluorescein derivatives with one alkylthio group in the 3 position replacing an -OH/-O^−^ group [51]. The origin of reduced fluorescence quantum yields in dyes with alkylthio donor groups in their chromophores is not clear at this point. It may result from enhanced internal conversion or intersystem crossing to the triplet state. Further photophysical work will have to settle this issue and thereby suggest structural improvements and/or the best applications of these dyes.

## Conclusion

The effective introduction of alkylthiol groups into the *para*-positions of triarylmethylium ions via S_N_Ar reactions was demonstrated. These new thioether-substituted triarylmethylium ions provide access to a broad range of new heterocyclic carbenium dyes of the xanthenium, acridinium and triangulenium type via further S_N_Ar reactions with primary amines and ring-closure reactions. The introduction of thioether donor groups in these dye classes is unprecedented, but is found to yield spectral properties quite similar to analogous dyes with dialkylamino groups. The synthesized thioether-substituted triangulenium derivatives are fluorescent, though with lower quantum yields (Φ_f_ = 0.1 to Φ_f_ = 0.3) than the corresponding dialkylamino-substituted analogues.

## Supporting Information

File 1 Experimental details, full synthetic procedures, spectroscopic characterization and NMR spectra of new compounds, as well as additional UV–vis and fluorescence spectra.
