# Supplementary material for: Synthesis and properties of sulfur-functionalized triarylmethylium, acridinium and triangulenium dyes
Source: Beilstein J Org Chem. 2019 Sep 9;15:2133–41. doi: 10.3762/bjoc.15.210 (PMC6753677; doi:10.3762/bjoc.15.210)
Supplement: File 1 — Experimental details, full synthetic procedures, spectroscopic characterization and NMR spectra of new compounds, as well as additional UV–vis and fluorescence spectra. [file Beilstein_J_Org_Chem-15-2133-s001.pdf]

BEILSTEIN JOURNAL OF ORGANIC CHEMISTRY

Supporting Information

for

Synthesis and properties of sulfur-functionalized triarylmethylium, acridinium and triangulenium dyes

Marco Santella, Eduardo Della Pia, Jakob Kryger Sørensen and Bo W. Laursen

Beilstein J. Org. Chem. **2019,** *15,* 2133–2141. doi:10.3762/bjoc.15.210

Experimental details, full synthetic procedures, spectroscopic characterization and NMR spectra of new compounds, as well as additional UV–vis and fluorescence spectra

License and Terms: This is a supporting information file under the terms of the Creative Commons Attribution License (http://creativecommons.org/licenses/by/4.0). Please note that the reuse, redistribution and reproduction in particular requires that the authors and source are credited.
The license is subject to the *Beilstein Journal of Organic Chemistry* terms and conditions: (https://www.beilstein-journals.org/bjoc)

Table of contents

Experimental Details S2

Synthetic procedures S3

NMR spectra S11

Additional spectroscopic data S23

References S23

Experimental Details

Chemicals were purchased from Sigma-Aldrich or Fluka and used as received. Solvents were of HPLC grade and used as received. $(TMP)_3C^+BF_4^-$, $(DMP)(TMP)_2C^+BF_4^-$ and $(DMP)_2(TMP)C^+BF_4^-$ were synthesized according to a literature procedure [1]. Thin layer chromatography was carried out using aluminum sheets precoated with silica gel 60 F_{254} (Merck 5554). Flash column chromatography was carried out using ROCC silica gel (40–63 µm). ^1H and ^{13}C NMR spectra were recorded on a 500 MHz instrument at 500 MHz and 126 MHz, respectively, with non-inverse cryoprobe using the residual solvent as the internal standard ($CDCl_3$ δ_H = 7.26 ppm, δ_C = 77.16 ppm, CD_3CN δ_H = 1.94ppm, δ_C = 1.32 ppm). All coupling constants are expressed in hertz (Hz). Mass spectrometry (MS) was performed using matrix assisted laser desorption ionization (MALDI) using dithranol as a matrix. Elemental analysis was performed at University of Copenhagen. Absorption spectra were recorded using a Cary 300 UV–vis from Agilent Technologies Cary. Fluorescence spectra were recorded using Cary Eclipse Fluorescence Spectrometer from Agilent Technologies. Time correlated single photon counting was performed using a Fluo Time 300, Fluorescence Lifetime Spectrometer form PicoQuant. Fluorescence quantum yields (QYs) were measured by multipoint determination using a Cary 300 UV–vis spectrophotometer for absorption measurements and a Fluotime 300 (PicoQuant)instrument for fluorescence measurements. Fluorescein in 0.1 M aqueous NaOH (Φ = 0.96) was used as reference dye. For all QY determinations data points were collected with a measured absorbance below 0.1 for the longest wavelength absorption to avoid inner filter effects. Calculations were done in ArgusLab[4] using semi-empirical method AM1.

Synthetic procedures

Tris(4-ethylthio-2,6-dimethoxyphenyl)methylium hexafluorophosphate (**1**)

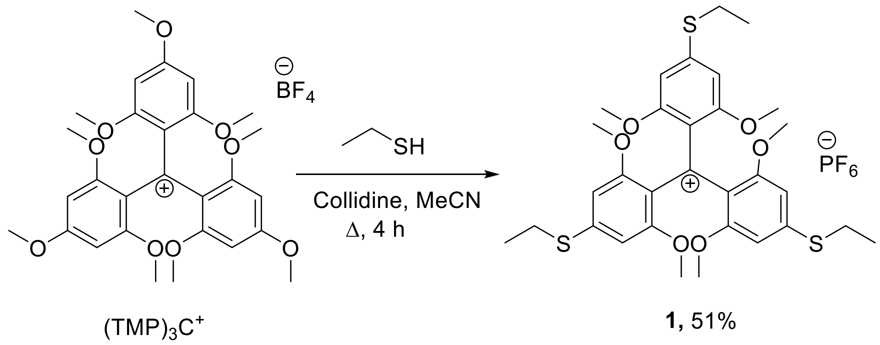

To a solution of $(TMP)_3C^+$ (4.00 g, 6.6 mmol) in 15 mL of MeCN, were added 19.5 mL of collidine and ethanethiol (16.57 g, 266.51 mmol). The reaction was stirred at reflux temperature and monitored by MALDI-TOF MS spectrometry until complete conversion of the starting material is observed, 4 h in total. The reaction mixture was then cooled to room temperature and poured onto 0.2 M KPF_6 (aq.) (200 mL), extracted with DCM (3 × 100 mL) and the combined organic phases were dried over $MgSO_4$. After filtration, the solvent volume was reduced in vacuum. To the remaining solution (collidine) was added heptane (100 mL) and the organic phase was removed by decantation. The sticky solid material was washed with heptane (3 × 100 mL). Column chromatography on silica gel (SiO_2) with dichloromethane/isopropanol 32:1 as eluent afforded the pure compound **1** as dark blue crystals (2.54 g, 51%). ^{1}H NMR (500 MHz, $CDCl_3$) δ 6.32 (s, 6H), 3.58 (s, 18H), 3.14 (q, *J* = 7.4 Hz, 6H), 1.42 (t, *J* = 7.4 Hz, 12H). ^{13}C NMR (126 MHz, $CDCl_3$) δ 166.81, 161.61, 156.09, 122.20, 101.66, 56.79, 26.35, 13.57. (MS) MALDI-TOF (dithranol matrix) m/z 603.24 [M^+]. Anal. Calcd. For $C_{31}H_{39}F_6O_6PS_3$, C: 49.87; H: 5.25; N: 0.0; Found C: 50.10; H: 5.55; N: 0.00.

(2,6-Dimethoxyphenyl)bis(4-ethylthio-2,6-dimethoxyohenyl)methylium hexafluorophosphate (**2**)

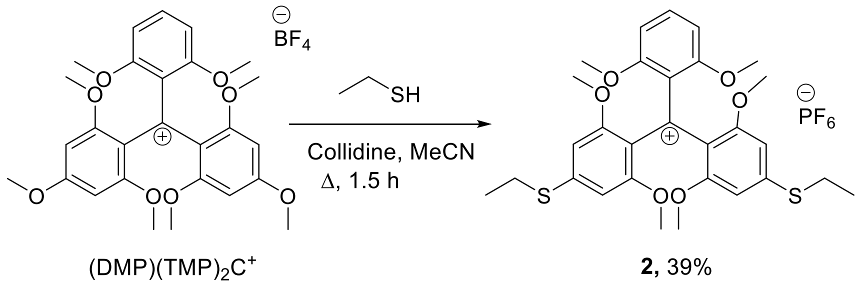

To a solution of $(DMP)(TMP)_2C^+$ (2.00 g, 3.51 mmol) in 15 mL of MeCN, were added 14 mL of collidine and ethanethiol (6.54 g, 105.2 mmol). The reaction was stirred at reflux temperature until MALDI-TOF MS spectrometry showed complete conversion of the starting material, 1.5 h in total. After cooling the solution to room temperature, the reaction mixture was poured onto 0.2 M KPF_6 (aq.) (200 mL). The organics were extracted with DCM (3 × 100 mL) and dried over $MgSO_4$. After filtration the solvent was reduced in vacuum. To the remaining solution (collidine) was added heptane (100 mL) and the organic phase was removed by decantation. The sticky solid material was washed with heptane (3 × 100 mL). The solid was reprecipitated from a mixture of DCM/heptane. Column chromatography on silica gel (SiO_2) with dichloromethane/isopropanol 45:1 as eluent afforded the

pure compound as blue solid (0.93g, 39%). ^{1}H NMR (500 MHz, $CDCl_3$) δ 7.31 (t, *J* = 8.4 Hz, 1H), 6.50 (d, *J* = 8.4 Hz, 2H), 6.32 (s, 4H), 3.59 (s, 12H), 3.57 (s, 6H), 3.22 – 3.14 (q, *J* = 7.4 Hz 4H), 1.44 (t, *J* = 7.4 Hz, 6H). ^{13}C NMR (126 MHz, $CDCl_3$) δ 162.55, 160.85, 159.08, 134.28, 123.49, 123.00, 104.56, 101.23, 56.82, 56.50, 26.38, 13.29. (MS) MALDI-TOF (dithranol matrix) m/z 543.49 [M^+]. Anal. Calcd. For $C_{29}H_{35}F_6O_6PS_2$, C: 50.58; H: 5.12; N: 0.0; Found C: 50.94; H: 5.15; N: 0.00.

Bis(2,6-Dimethoxyphenyl)(4-ethylthio-2,6-dimethoxyphenyl)methylium hexafluorophosphate (**3**)

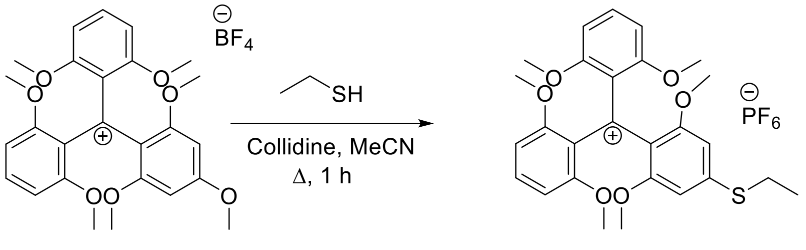

To a solution of $(DMP)_2(TMP)C$ (0.50g, 0.9 mmol) in 5 mL of MeCN, were added 2 mL of collidine and ethanethiol (0.87 g, 13.88 mmol). The reaction was stirred at reflux temperature until MALDI-TOF MS spectrometry showed complete conversion of the starting material, 1 h in total. The reaction mixture was cooled to room temperature and poured onto 0.2 M KPF_6 (aq.) (200 mL). The reaction mixture was extracted with DCM (3 × 100 mL) and the combined organic phases were dried over $MgSO_4$. After filtration the solvent volume was reduced in vacuum. To the remaining solution (collidine) was added heptane (100 mL) and the organic phase was removed by decantation. The sticky solid material was washed with heptane (3 × 100 mL). Column chromatography on silica gel (SiO_2) with DCM: *iso*-propanol 43:1 as eluent afforded the pure material (0.12 g 21%) as a dark solid **3**. ^{1}H NMR (500 MHz, $CDCl_3$) δ 7.32 (t, *J* = 8.4 Hz, 2H), 6.50 (d, *J* = 8.4 Hz, 4H), 6.31 (s, 2H), 3.63 (s, 6H), 3.56 (s, 12H), 3.36 (q, *J* = 7.4 Hz, 2H), 1.48 (t, *J* = 7.4 Hz, 3H). ^{13}C NMR (126 MHz, $CDCl_3$) δ 175.05, 170.64, 165.88, 159.18, 134.61, 126.77, 123.46, 104.68, 100.67, 57.47, 56.60, 27.11, 13.02. (MS) MALDI-TOF (dithranol matrix) m/z 483.42 [M^+]. Anal. Calcd. For $C_{27}H_{31}F_6O_6PS$, C: 51.59; H: 4.97; N: 0.0; Found C: 51.54; H: 4.77; N: 0.00.

9-(4-Ethylthio-2,6-dimethoxyphenyl)-10-propyl-2,7-bis(ethylthio)-4,5-dimethoxyacridinium hexafluorophosphate (**4a**)

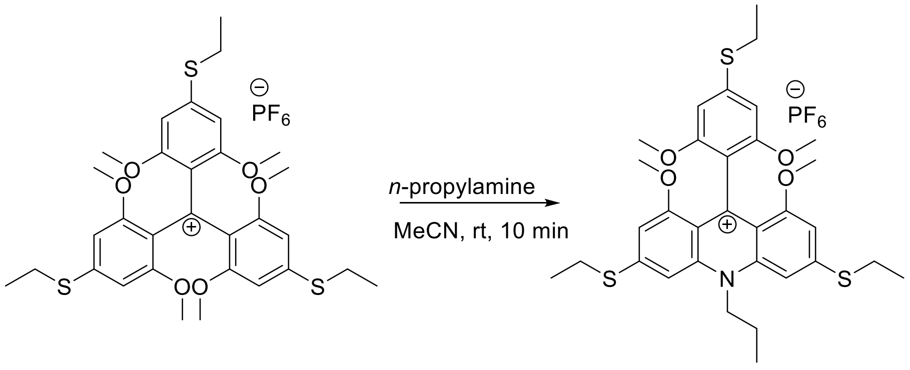

1 **4a**, 59%

In a 100 mL round-bottomed flask, compound **1** (210 mg, 0.28 mmol) was dissolved in 10 mL of MeCN, and *n*-propylamine (33 mg, 0.56 mmol) was subsequently added. The mixture was stirred at room temperature changing immediately color from blue to red. After 10 minutes the reaction was monitored by MALDI-TOF MS spectrometry and the only presence of the product was observed. The reaction mixture was poured onto 0.2 M KPF_6 (100 mL). The crude material was collected by filtration and washed with H_2O (20 mL) and heptane (20 mL). Recrystallization from iPrOH with addition of few drop of MeCN to help the solubilization afforded the pure compound (123 mg, 59%) as dark brown crystals. ^{1}H NMR (500 MHz, CD_3CN) δ 7.28 (s, 2H), 6.83 (s, 2H), 6.72 (s, 2H), 4.80 – 4.75 (m, 2H), 3.56 (s, 6H), 3.52 (s, 6H), 3.30 (q, J = 7.4 Hz, 4H), 3.09 (q, J = 7.3 Hz, 2H), 2.16 (m, 2H), 1.47 (t, J = 7.4 Hz, 6H), 1.37 (t, J = 7.3 Hz, 3H), 1.25 (t, J = 7.4 Hz, 3H). ^{13}C NMR (126 MHz, CD_3CN) δ 160.62, 156.52, 154.22, 142.50, 138.73, 118.01, 105.43, 105.37, 103.45, 57.68, 56.74, 53.68, 28.25, 26.34, 21.40, 14.86, 13.68, 11.19. (2 signal missing, overlap). (MS) MALDI-TOF (dithranol matrix) m/z 598.41 [M^+]. Anal. Calcd. For $C_{32}H_{40}F_6NO_4PS_3$, C: 51.67; H: 5.42; N: 1.88; Found C: 51.82; H: 5.26; N: 1.81.

9-(4-Ethylthio-2,6-dimethoxyphenyl)-10-octyl-2,7-bis(ethylthio)-4,5-dimethoxyacridinium hexafluorophosphate (**4b**)

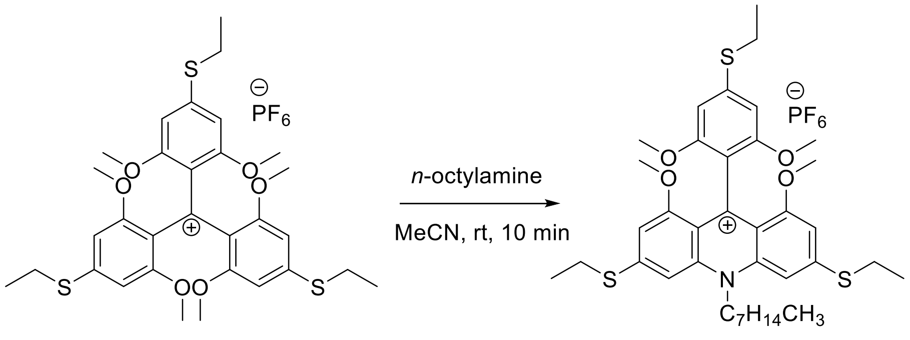

1 **4b**, 33%

In a 100 mL round-bottomed flask, compound **1** (210 mg, 0.28 mmol) was dissolved in 10 mL of acetonitrile, and *n*-octylamine (73 mg, 0.56 mmol) was added. The mixture was stirred at room temperature. The reaction mixture changed immediately color from blue to red. After 10 minutes MALDI-TOF MS spectrometry showed the only presence of the product. The reaction mixture was poured onto 0.2 M KPF_6 (100 mL). The crude material was collected by filtration and washed with H_2O (20 mL) and heptane (20 mL) and dried. Recrystallization from iPrOH with addition of few drop of MeCN to help the solubilization afforded the pure compound **4b** (75 mg, 33%.) as orange crystals. ^1H NMR (500 MHz, CD_3CN) δ 7.27 (s, 2H), 6.83 (s, 2H), 6.72 (s, 2H), 4.84 – 4.78 (m, 2H), 3.56 (s, 6H), 3.52 (s, 6H), 3.29 (q, *J* = 7.3 Hz, 4H), 3.10 (q, *J* = 7.3 Hz, 2H), 2.13 – 2.06 (m, 2H), 1.71 – 1.65 (m, 2H), 1.55 – 1.48 (m, 3H), 1.46 (t, *J* = 7.3 Hz, 6H), 1.43 – 1.32 (m, 8H), 0.92 (t, *J* = 6.8 Hz, 3H). ^{13}C NMR (126 MHz, CD_3CN) δ 160.60, 156.48, 154.17, 142.47, 138.74, 118.02, 105.48, 105.37, 103.36, 57.69, 56.75, 52.45, 32.47, 29.91, 29.88, 28.26, 27.83, 27.26, 26.35, 23.33, 14.86, 14.37, 13.73(2 signal missing, overlap). (MS) MALDI-TOF (dithranol matrix) m/z 668.43 [M^+]. Anal. Calcd. For $C_{37}H_{50}F_6NO_4PS_3$, C: 54.60; H: 6.19; N: 1.72; Found C: 54.68; H: 6.00; N: 1.67.

2,6,10-Tris(ethylthio)-4-propyl-4-aza-8,12-dioxatriangulenium hexafluorophosphate (**5a**)

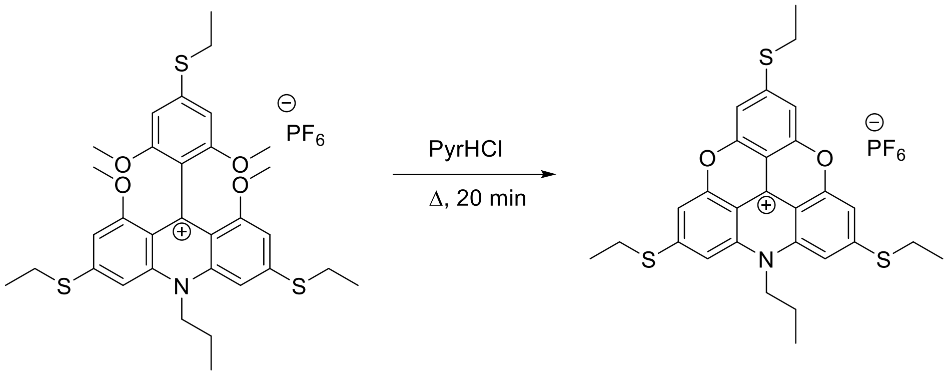

4a **5a**, 75%

In a 100 mL Erlenmeyer flask, pyridinium hydrochloride (3.30 g, 28.6 mmol) was heated at 200 °C and then compound **4a** (150 mg, 0.20 mmol) was added as solid in one portion. After 20 minutes MALDI-TOF MS spectrometry showed presence of only the product. To the reaction mixture was added a solution of 0.2 M KPF_6 (60 mL). The crude material was collected by filtration and washed with H_2O (20 mL) and heptane (20 mL). The crude material was purified by column chromatography on silica gel (SiO_2) with DCM/isopropanol 40:1 as eluent, affording **5a** as orange solid **(**131mg, 75%**)**. ^1H NMR (500 MHz, CD_3CN) δ 7.18 (s, 2H), 7.10 (s, 2H), 7.04 (s, 2H), 4.39 – 4.28 (m, 2H), 3.26 (q, *J* = 7.4 Hz, 4H), 3.17 (q, *J* = 7.4 Hz, 2H), 1.88 (m, 2H), 1.47 (t, *J* = 7.4 Hz, 6H), 1.43 (t, *J* = 7.4 Hz, 3H), 1.14 (t, *J* = 7.4 Hz, 3H).^{13}C NMR (126 MHz, CD_3CN) δ 156.78, 155.05, 151.81, 150.86, 139.17, 135.13, 107.18, 105.81, 104.74, 104.35, 101.09, 99.66, 48.48, 25.62, 19.11, 12.24, 9.84 (one signal missing). (MS) MALDI-TOF (dithranol matrix) m/z 506.04 [M^+]. Anal. Calcd. For $C_{28}H_{28}F_6NO_2PS_3$, C: 51.60; H: 4.33; N: 2.15; Found C: 51.43; H: 4.15; N: 2.41.

2,6,10-Tris(ethylthio)-4-octyl-4-aza-8,12-dioxatriangulenium hexafluorophosphate (**5b**)

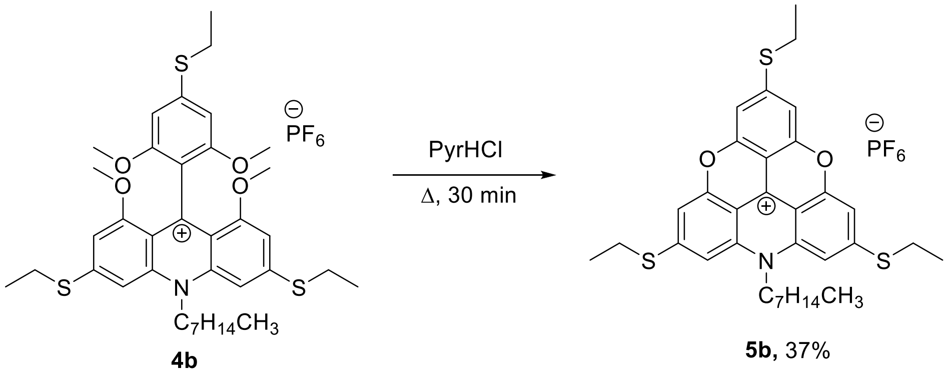

In a 100 mL Erlenmeyer flask, pyridinium hydrochloride (3.40 g, 29,4 mmol) was heated at 220 °C and subsequently compound **4b** (170 mg, 0.21 mmol) was added as solid. After 30 minutes MALDI-TOF MS spectrometry showed the only presence of the product. To the reaction mixture was added a solution of 0.2 M KPF_6 (80 mL). The crude material was collected by filtration and washed with H_2O (20 mL) and heptane (20 mL). The crude material was purified by column chromatography on silica gel (SiO_2) with DCM/isopropanol 40:1 as eluent affording **5b** as red powder after reprecipitation from DCM/*n*-heptane (66 mg, 37%.). ^{1}H NMR (500 MHz, , CD_3CN) δ 7.17 (s, 2H), 7.12 (s, 2H), 7.06 (s, 2H), 4.40 – 4.34 (m, 2H), 3.27 (q, *J* = 7.4 Hz, 4H), 3.18 (q, *J* = 7.4 Hz, 2H), 1.84 (m, 2H), 1.60 – 1.54 (m, 2H), 1.47 (t, *J* = 7.4 Hz, 6H), 1.43 (t, *J* = 7.4 Hz, 3H), 1.34 (m, 8H), 0.91 (t, *J* = 6.8 Hz, 3H). ^{13}C NMR (126 MHz, CD_3CN) δ 157.96, 156.28, 153.18, 152.26, 140.51, 136.55, 108.56, 107.20, 105.97, 105.79, 102.53, 48.57, 32.54, 29.97, 27.14, 26.92, 26.85, 23.38, 14.41, 13.61, 13.57(2 signal missing, overlap). (MS) MALDI-TOF (dithranol matrix) m/z 576.54 [M^+]. Anal. Calcd. For $C_{33}H_{38}F_6NO_2PS_3$, C: 54.91; H: 5.31; N: 1.94; Found C: 55.14; H: 5.13; N: 1.79.

Tris(ethylthio)trioxatriangulenium hexafluorophosphate (**6**)

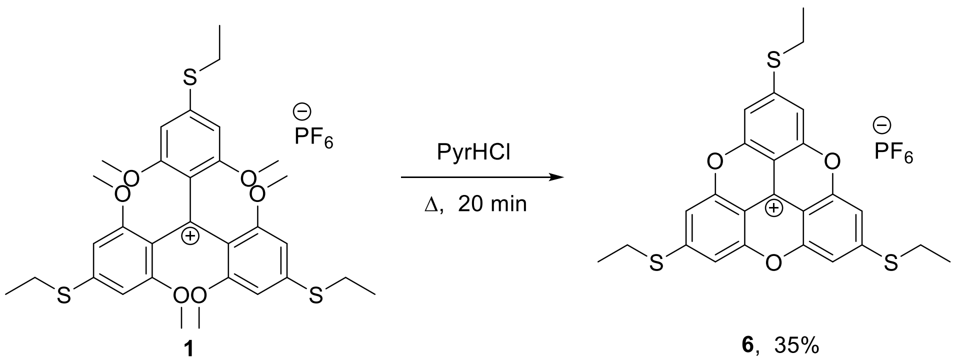

In a 100 mL Erlenmeyer flask, pyridinium hydrochloride (1.35g, 11.7 mmol) was heated at 220 °C and then subsequently **1** (350 mg, 0.47 mmol) was added as solid. The reaction mixture changed immediately color from blue to red. After 20 minutes MALDI-TOF MS spectrometry showed the only presence of the product and the heating was stopped. To the reaction mixture was added 0.2 M KPF_6 (100 mL). The crude material was collected by filtration and washed with H_2O (20 mL) and

heptane (20 mL). The material was purified by column chromatography on silica gel (SiO_2) with DCM/methanol 40:1 as eluent. The pure material (**6**) was obtained after recrystallization from iPrOH/MeCN (75 mg, 35%) as red powder. ^{1}H NMR (500 MHz, CD_3CN) δ 7.34 (s, 6H), 3.25 (q, J = 7.4 Hz, 6H), 1.45 (t, J = 7.4 Hz, 9H). ^{13}C NMR (126 MHz, CD_3CN) δ 161.01, 153.25, 138.94, 109.33, 103.23, 27.16, 13.48. (MS) MALDI-TOF (dithranol matrix) m/z 465.22 [M^+]. Anal. Calcd. For $C_{25}H_{21}F_6O_3PS_3$, C: 49.18; H: 3.47; N: 0.00; Found C: 49.13; H: 3.21; N: 0.00.

9-(2,6-Dimethoxyphenyl)-2,7-bis(ethylthio)-4,5-dimethoxyxanthenium hexafluorophosphate (**7**)

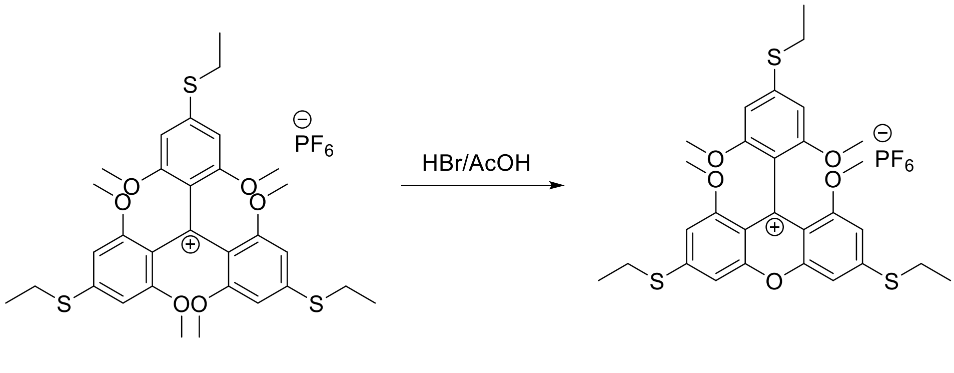

1 **7** 43%

In a 100 mL round-bottomed flask, compound **1** (235 mg, 0.31 mmol) was dissolved in AcOH (20 mL), and subsequently 10 mL of 48% aqueous HBr were added. The mixture was heated to reflux for 3.5 h until MALDI-TOF MS spectrometry analysis showed that the reaction was completed. After that, the reaction mixture was cooled and poured onto 0.2 M KPF_6 (100 mL). The crude material was collected by filtration and washed with H_2O (20 mL) and heptane (20 mL). Recrystallization from iPrOH yielded pure **7** as green crystals (93 mg, 43%). ^{1}H NMR (500 MHz, CD_3CN) δ 7.26 (d, J = 1.7 Hz, 2H), 6.86 (d, J = 1.7 Hz, 2H), 6.72 (s, 2H), 3.62 (s, 6H), 3.58 (s, 6H), 3.27 (q, J = 7.4 Hz, 4H), 3.11 (q, J = 7.3 Hz, 2H), 1.44 (t, J = 7.4 Hz, 6H), 1.37 (t, J = 7.3 Hz, 3H). ^{13}C NMR (126 MHz, CD_3CN) δ 197.17, 162.65, 161.90, 159.82, 155.35, 155.30, 139.83, 113.95, 105.19, 103.81, 103.62, 56.82, 55.62, 26.56, 25.47, 13.42, 12.32. (MS) MALDI-TOF (dithranol matrix) m/z 557.39 [M^+]. Anal. Calcd. For $C_{31}H_{39}F_6N_2O_5P$, C: 56.02; H: 4.46; N: 0.00, Found C: 49.48; H: 6.05; N: 3.89.

Bis(ethylthio)trioxatriangulenium hexafluorophosphate (**8**)

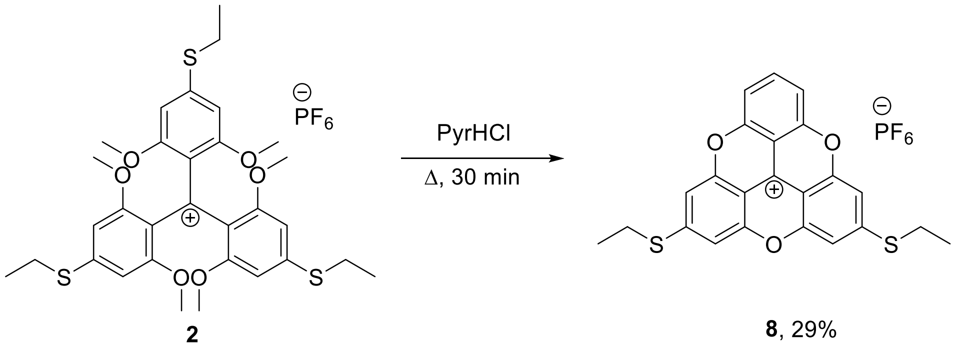

In a 100 mL Erlenmeyer flask, pyridinium hydrochloride (5.0 g, 43.20 mmol) was heated at 220 °C and subsequently compound **2** (350 mg, 0.47 mmol) was added as a solid. The reaction mixture changed immediately color from blue to red. After 30 minutes MALDI-TOF MS spectrometry showed the only presence of the product. The reaction mixture was poured onto 0.2 M KPF_6 (100 mL). The crude material was collected by filtration and washed with H_2O (20 mL) and heptane (20 mL). The material was purified by column chromatography on silica gel (SiO_2) with DCM/iPrOH 20:1 as eluent affording the desired product (80 mg, 29%.) as pale orange crystals. ^{1}H NMR (500 MHz, CD_3CN) δ 8.30 (t, *J* = 8.5 Hz, 1H), 7.65 (d, *J* = 8.5 Hz, 2H), 7.51 (dd, *J* = 11.3, 1.0 Hz, 4H), 3.32 (q, *J* = 7.4 Hz, 4H), 1.49 (t, *J* = 7.4 Hz, 6H). ^{13}C NMR (126 MHz, CD_3CN) δ 160.64, 152.58, 152.42, 152.39, 141.39, 140.06, 112.17, 108.06, 107.99, 102.92, 25.80, 12.21. (MS) MALDI-TOF (dithranol matrix) m/z 405.21 [M^+]. Anal. Calcd. For $C_{23}H_{17}F_6O_3PS_2$, C: 50.18; H: 3.11 N: 0.00; Found C: 49.71; H: 2.83; N: 0.00.

2-Ethylthiotrioxatriangulenium hexafluorophosphate (**9**)

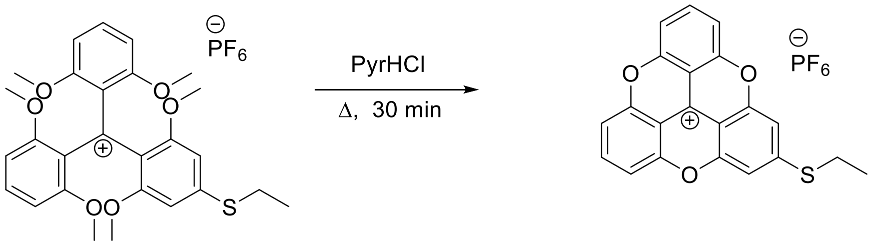

In a 100 mL Erlenmeyer flask, pyridinium hydrochloride (3.0 g, 25.80 mmol) was heated at 220 °C and subsequently **3** (90 mg, 0.14 mmol) was added as a solid. The reaction mixture changed immediately color from blue to red. After 30 minutes MALDI-TOF MS spectrometry showed the only presence of the product. The resulting mixture was poured onto 0.2 M KPF_6 (100 mL). The crude material was collected by filtration and washed with H_2O (20 mL) and heptane (20 mL). The material was purified by column chromatography on silica gel (SiO_2) with DCM/iPrOH 20:1 as eluent. Recrystallization from iPrOH yielded pure **9** (32 mg, 46%.) as red crystals. ^{1}H NMR (500 MHz, CD_3CN) δ 8.36 (t, *J* = 8.5 Hz, 2H), 7.71 (d, *J* = 8.6 Hz, 2H), 7.69 (d, *J* = 8.6 Hz, 2H), 7.58 (s, 2H), 3.33 (q, *J* = 7.4 Hz, 2H), 1.47 (t, *J* = 7.4 Hz, 3H). ^{13}C NMR (126 MHz, CD_3CN) δ 163.58,

154.24, 154.22, 154.03, 143.69, 143.49, 113.62, 113.38, 109.33, 106.61, 105.09, 27.20, 13.52. (MS) MALDI-TOF (dithranol matrix) m/z 345.03 [M^+]. Anal. Calcd. For $C_{21}H_{13}F_6O_3PS$, C: 51.44; H: 2.67 N: 0.00; Found C: 51.19; H: 2.35; N: 0.00.

NMR spectra

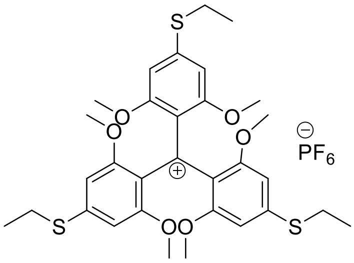

1

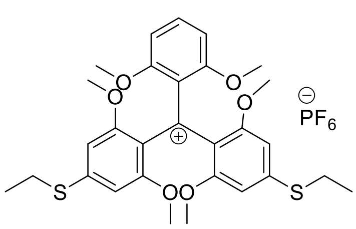

2

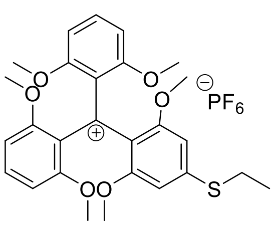

3

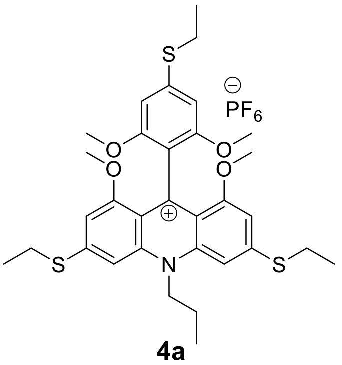

4a

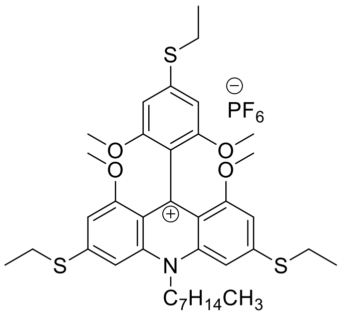

4b

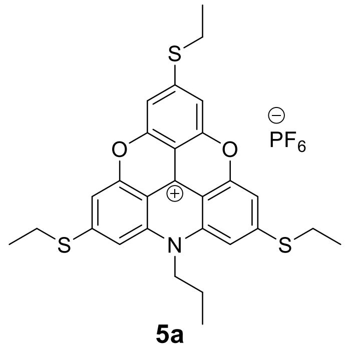

5a

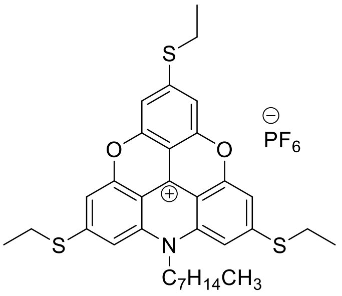

5b

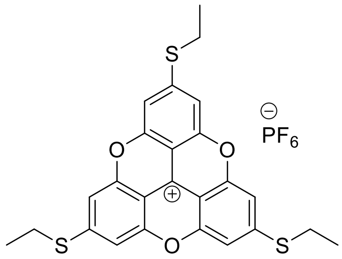

6

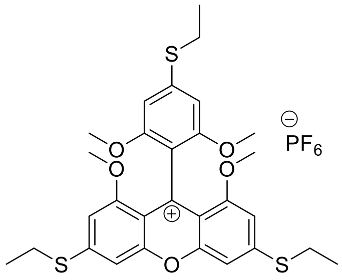

7

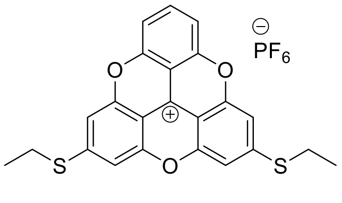

8

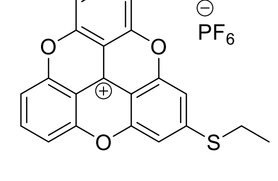

9

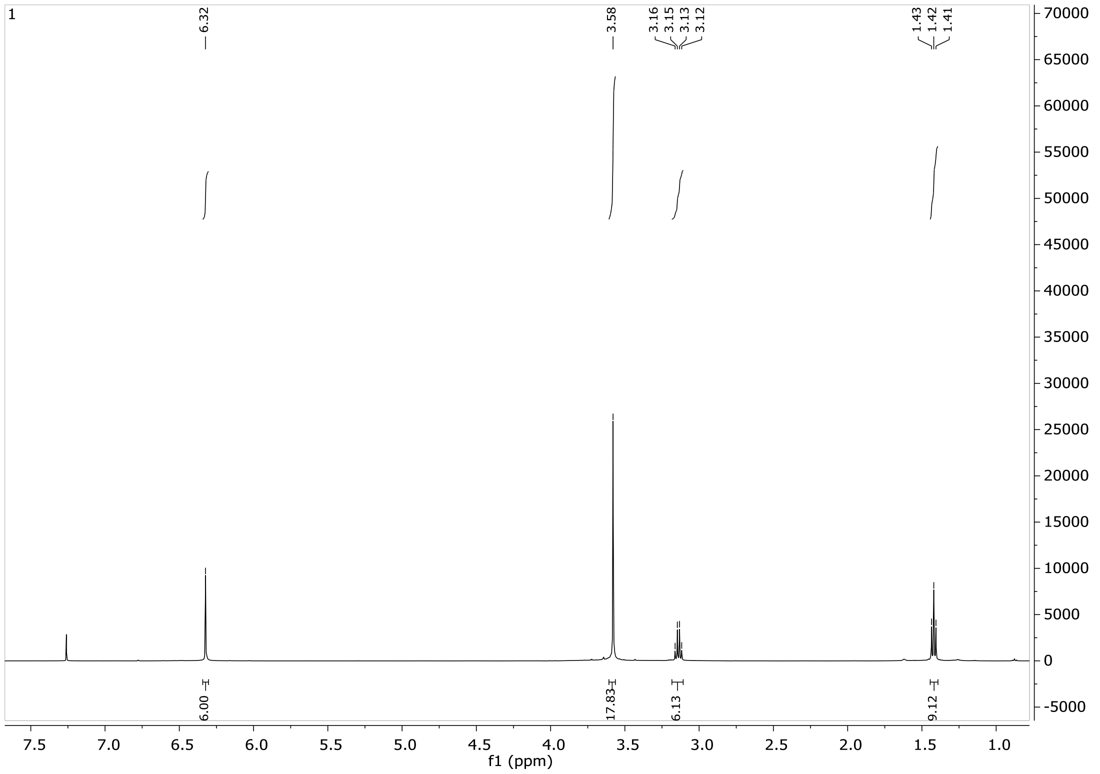

^{1}H NMR (500 MHz) spectrum of compound **1** recorded in $CDCl_3$.

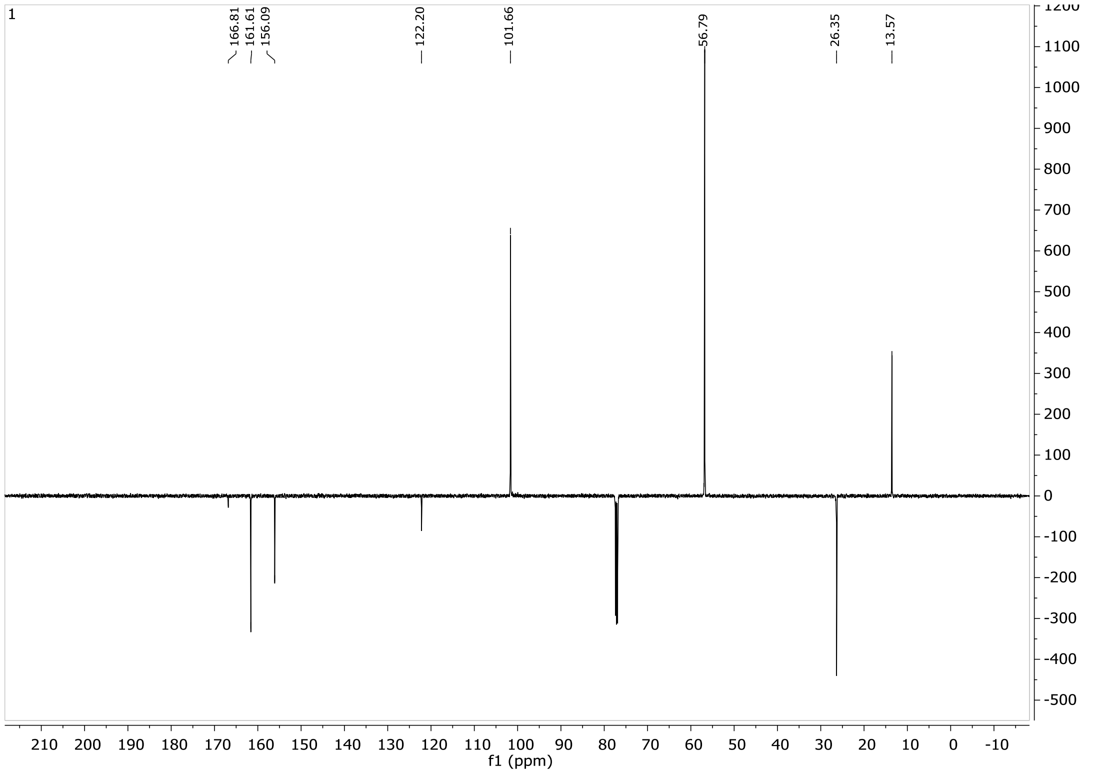

^{13}C APT NMR (126 MHz) spectrum of compound **1** recorded in $CDCl_3$.

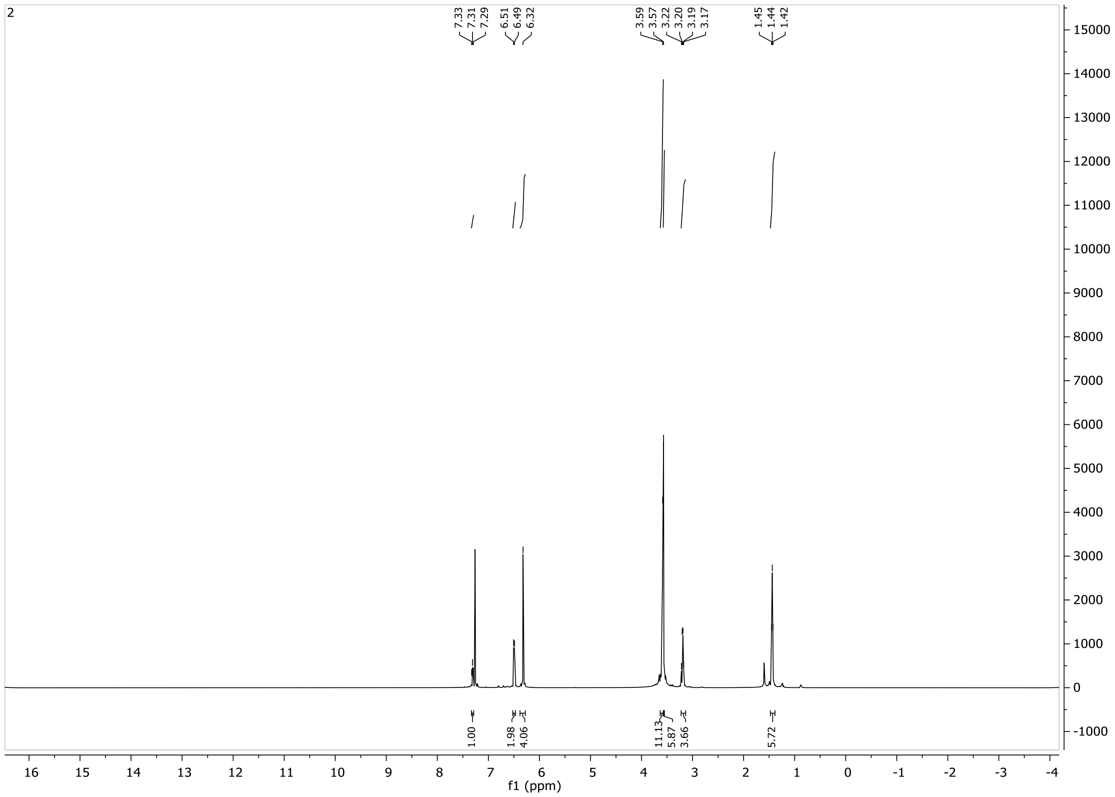

^{1}H NMR (500 MHz) spectrum of compound **2** recorded in $CDCl_3$.

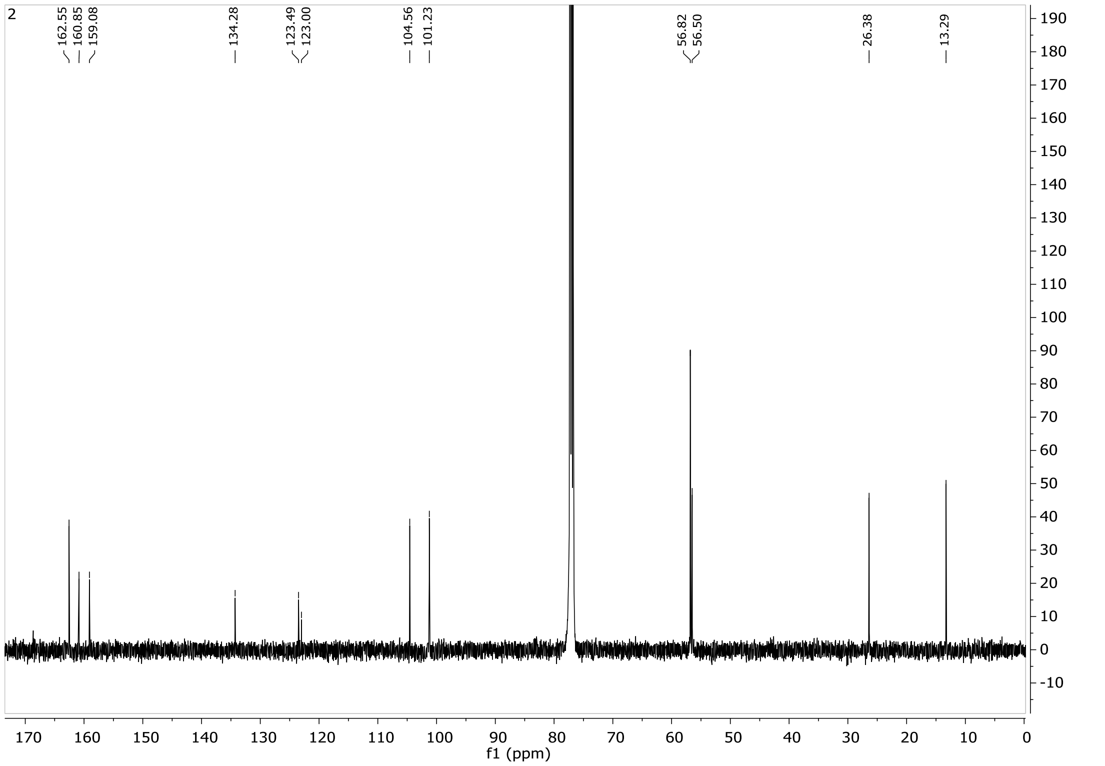

^{13}C NMR (126 MHz) spectrum of compound **2** recorded in $CDCl_3$.

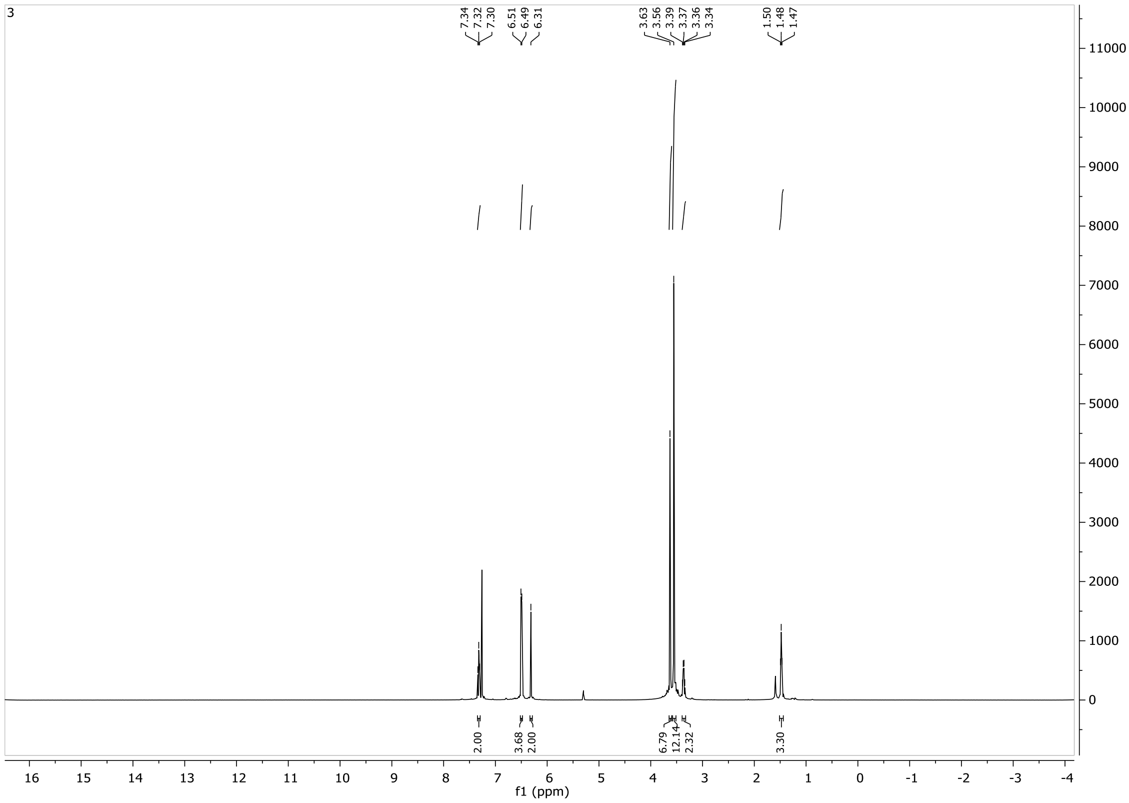

^{1}H NMR (500 MHz) spectrum of compound **3** recorded in $CDCl_3$.

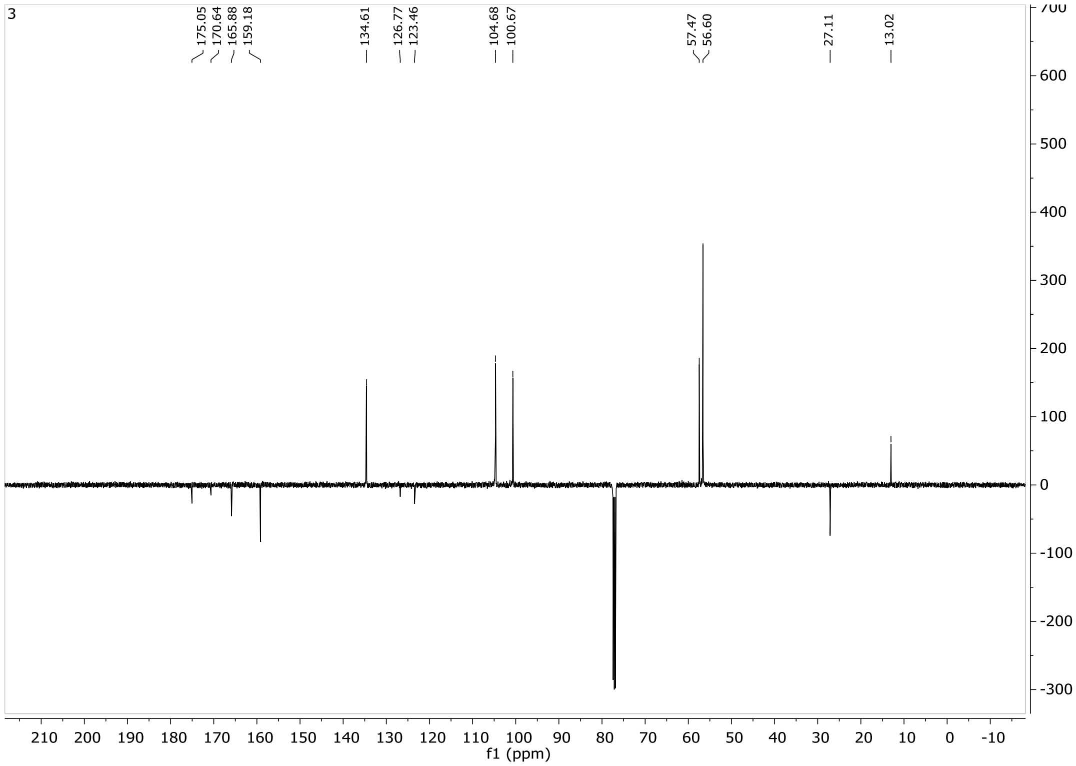

^{13}C APT NMR (126 MHz) spectrum of compound **3** recorded in $CDCl_3$.

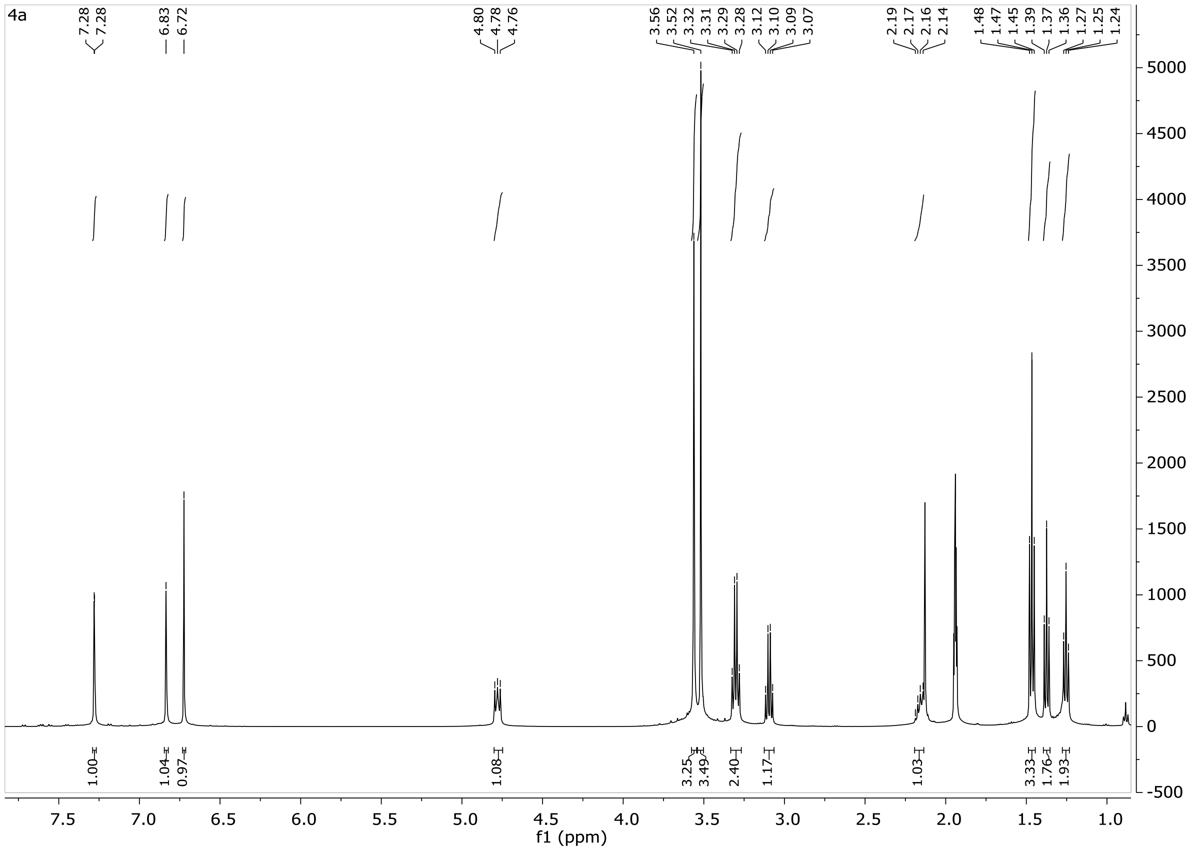

^{1}H NMR (500 MHz) spectrum of compound **4a** recorded in CD_3CN.

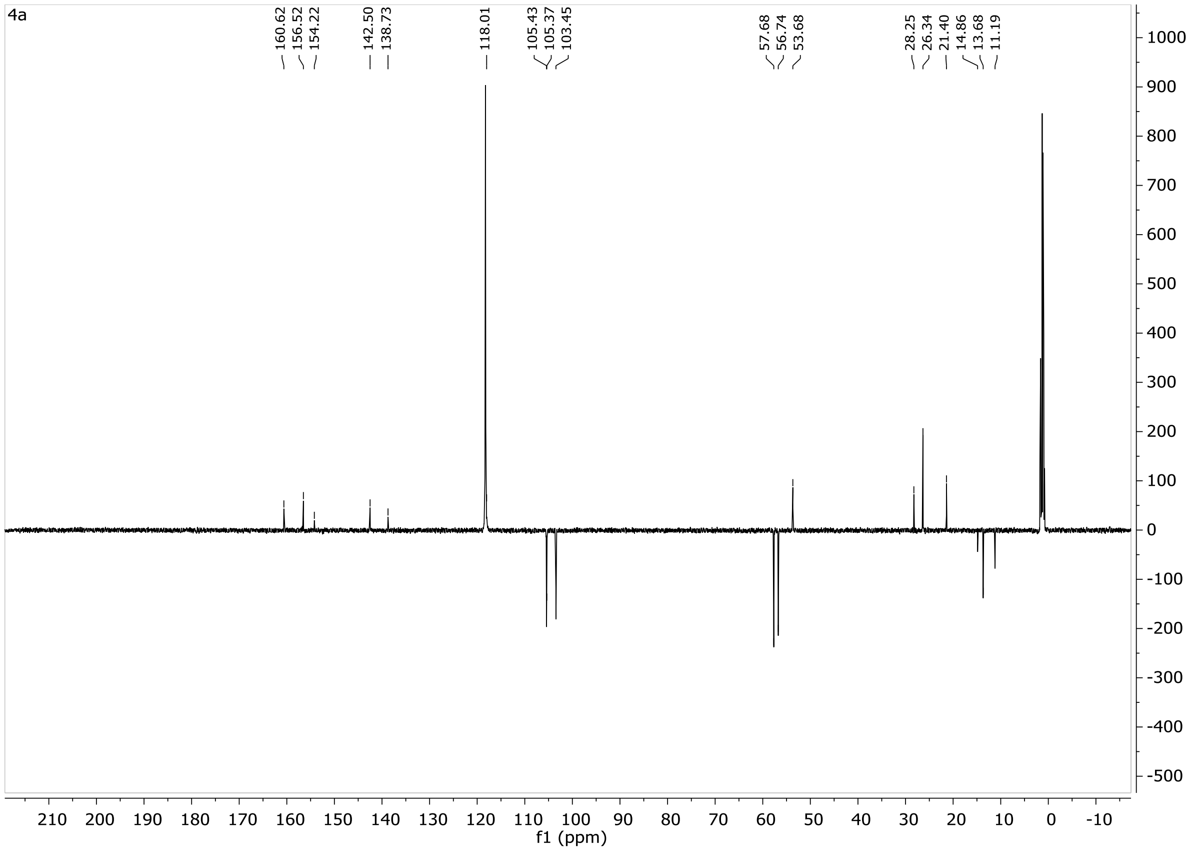

^{13}C APT NMR (126 MHz) spectrum of compound **4a** recorded in CD_3CN.

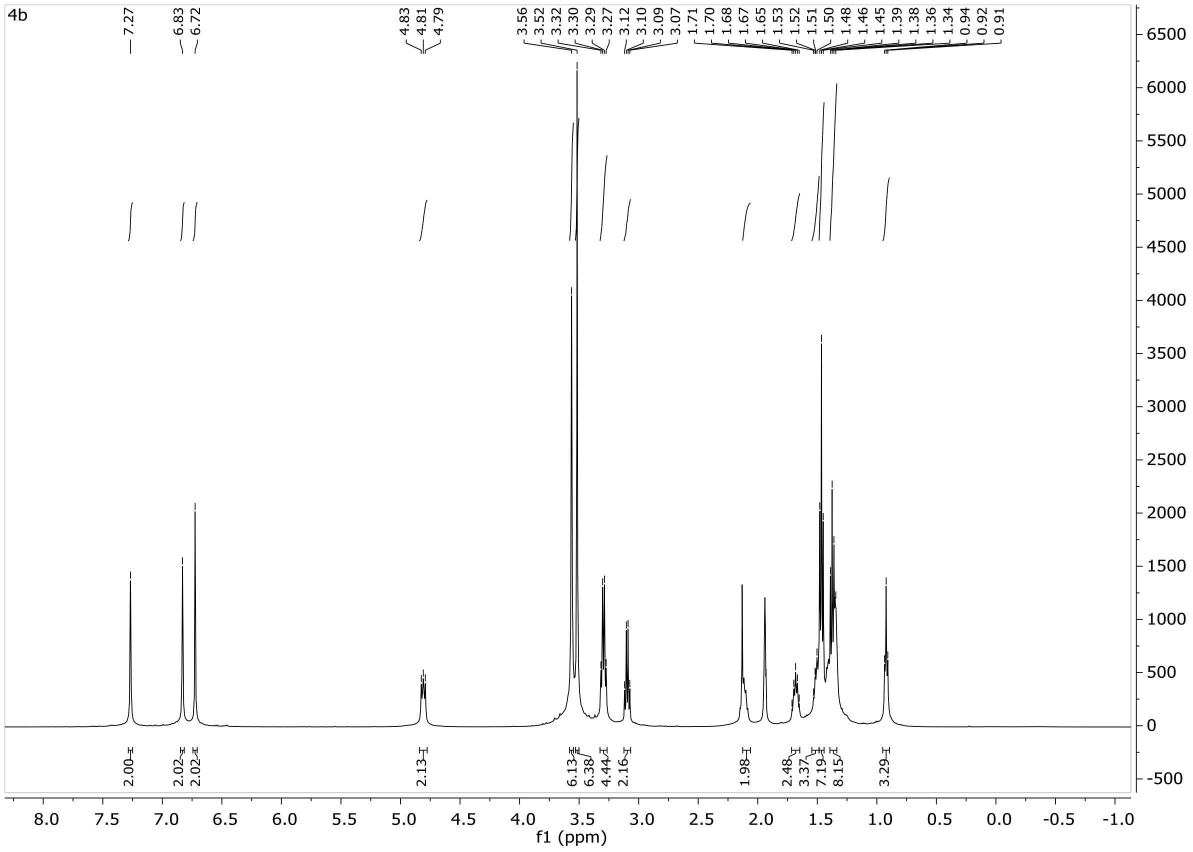

^{1}H NMR (500 MHz) spectrum of compound **4b** recorded in CD_3CN.

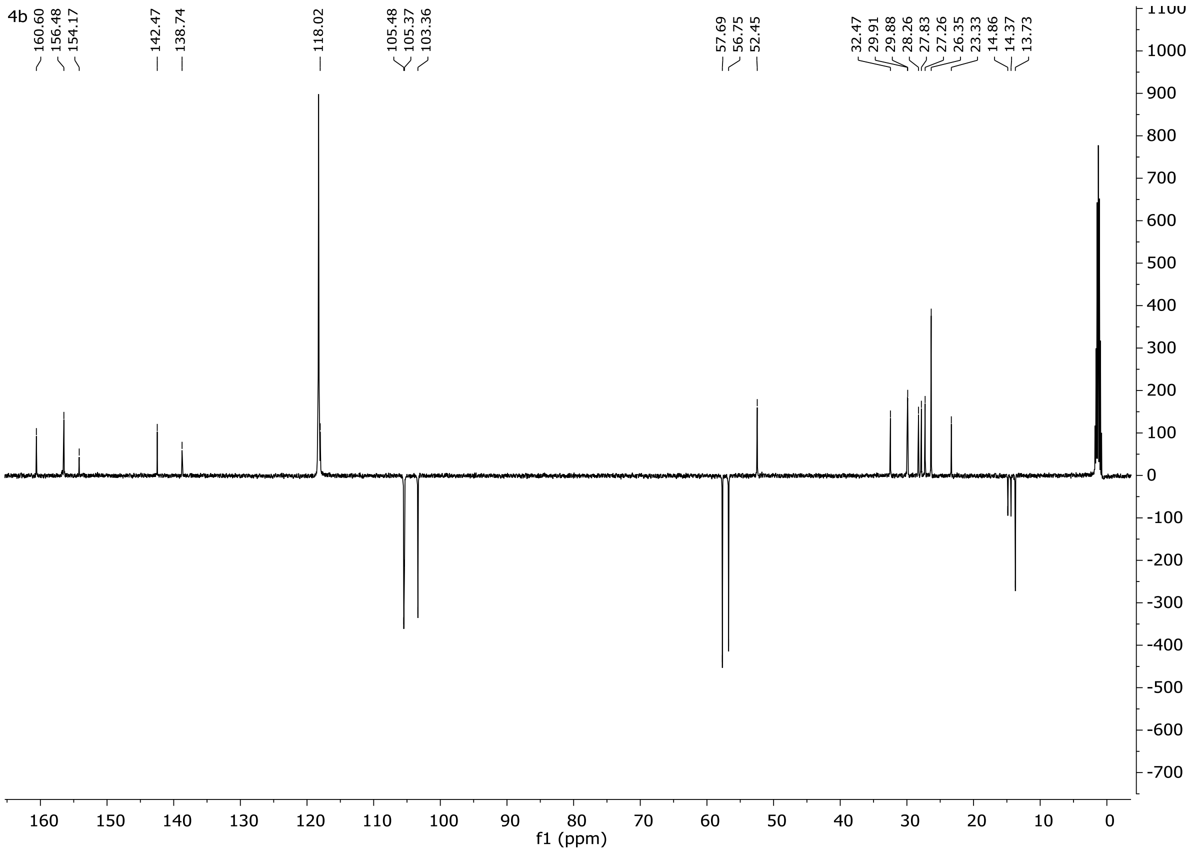

^{13}C APT NMR (126 MHz) spectrum of compound **4b** recorded in CD_3CN.

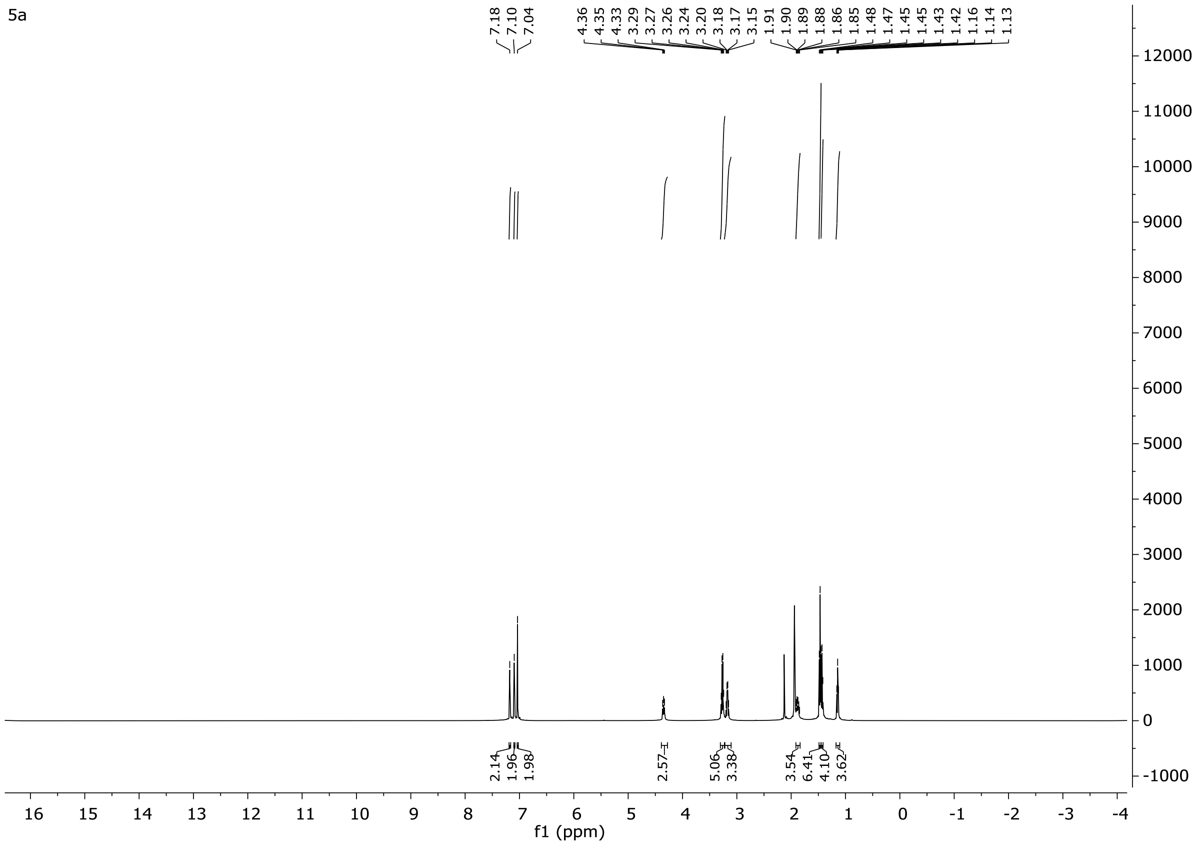

^{1}H NMR (500 MHz) spectrum of compound **5a** recorded in CD_3CN.

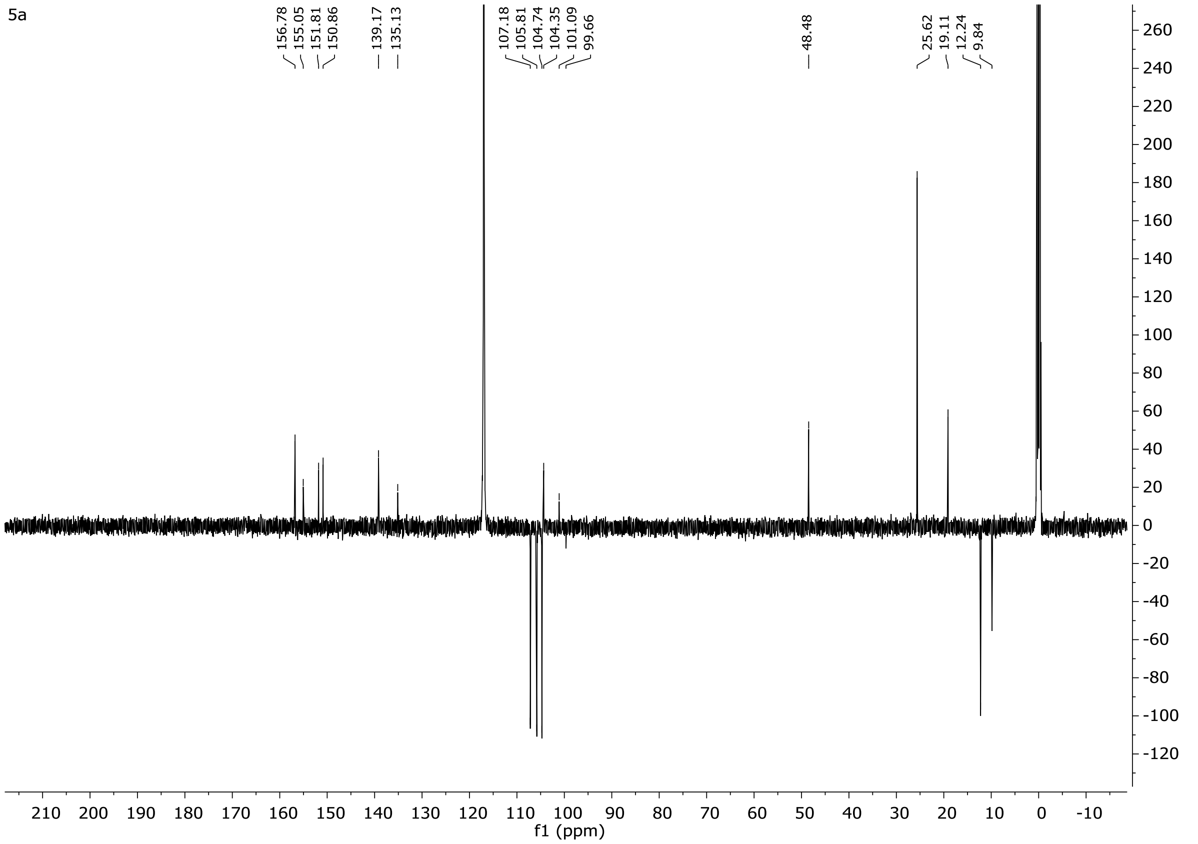

^{13}C APT NMR (126 MHz) spectrum of compound **5a** recorded in CD_3CN.

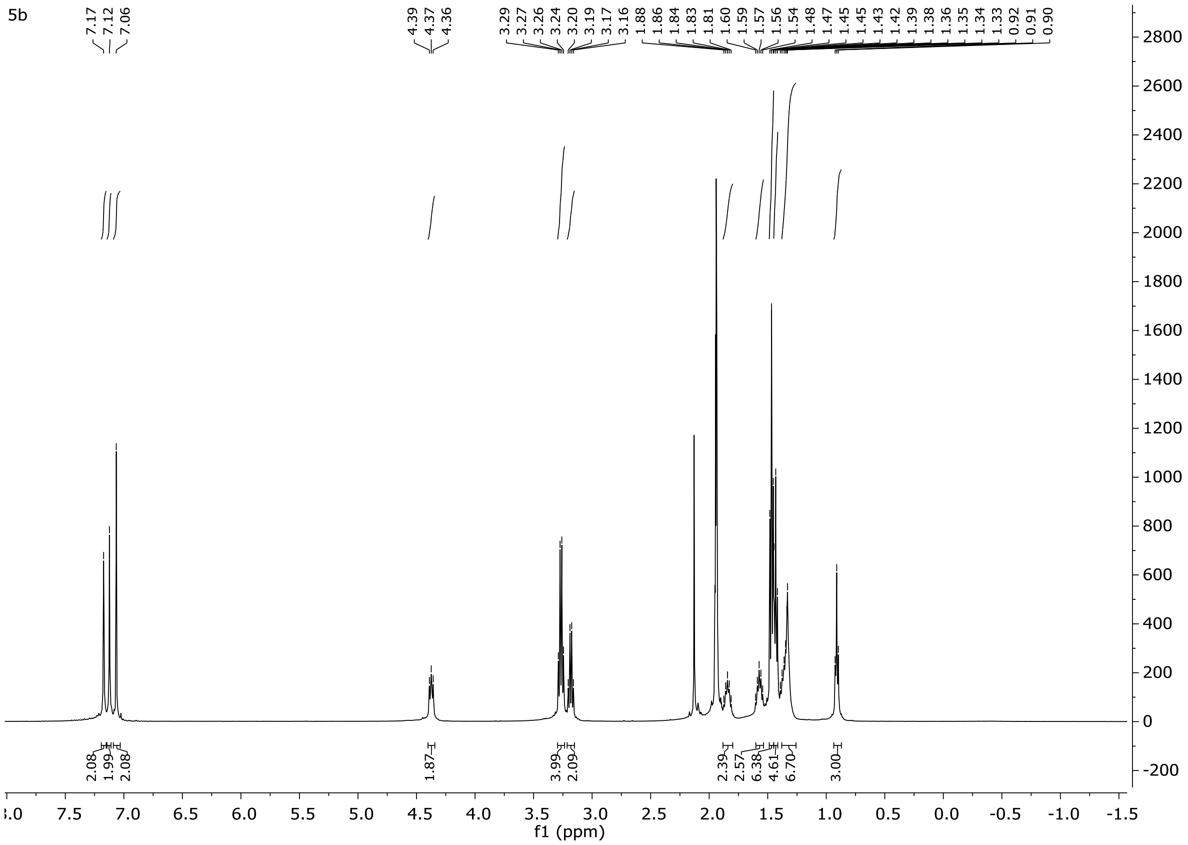

^{1}H NMR (500 MHz) spectrum of compound **5b** recorded in CD_3CN.

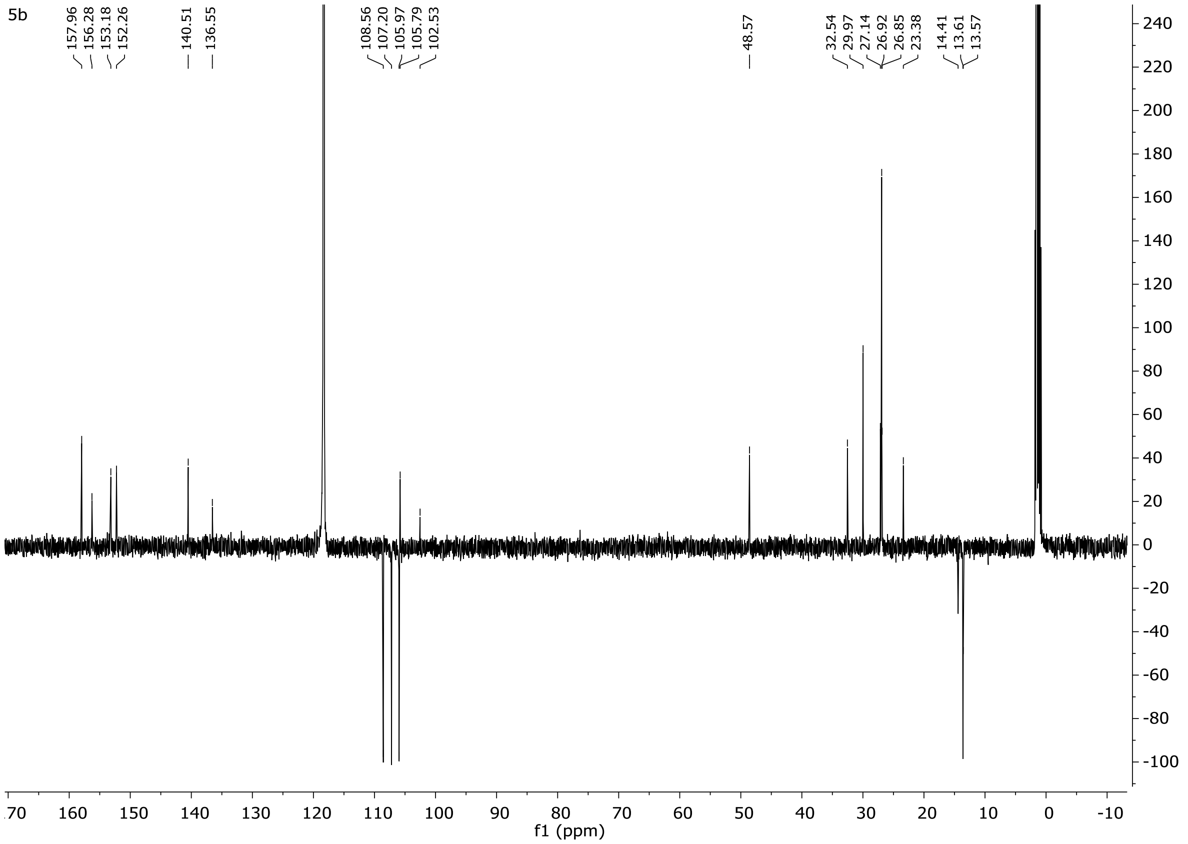

^{13}C APT NMR (126 MHz) spectrum of compound **5b** recorded in CD_3CN.

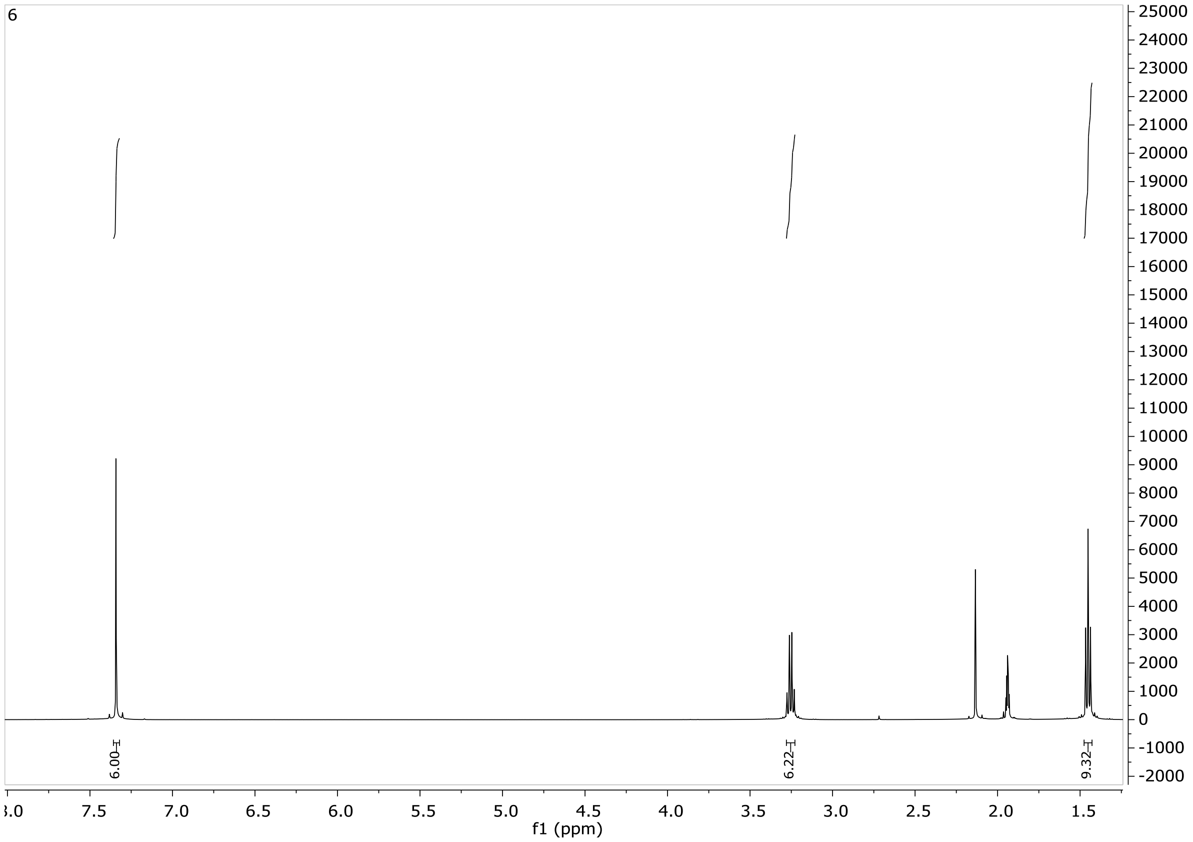

^{1}H NMR (500 MHz) spectrum of compound **6** recorded in CD_3CN.

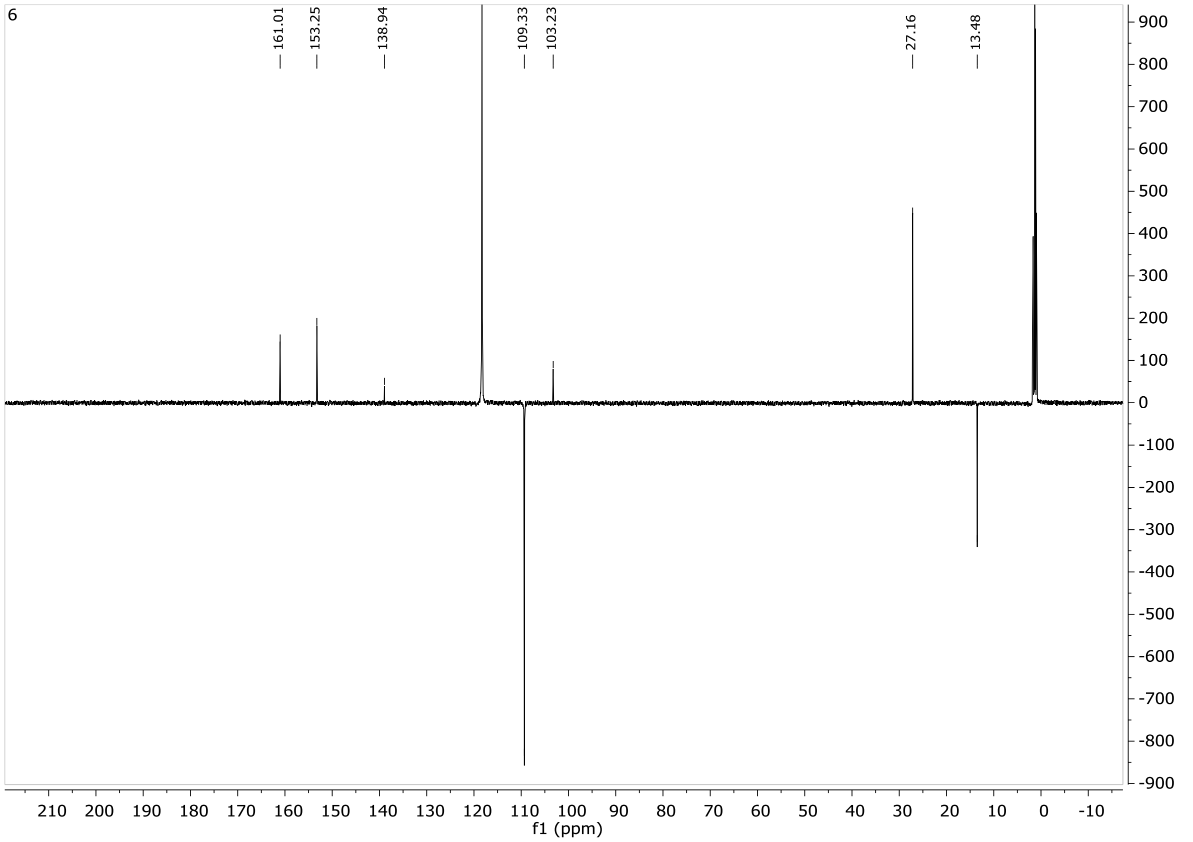

^{13}C APT NMR (126 MHz) spectrum of compound **6** recorded in CD_3CN.

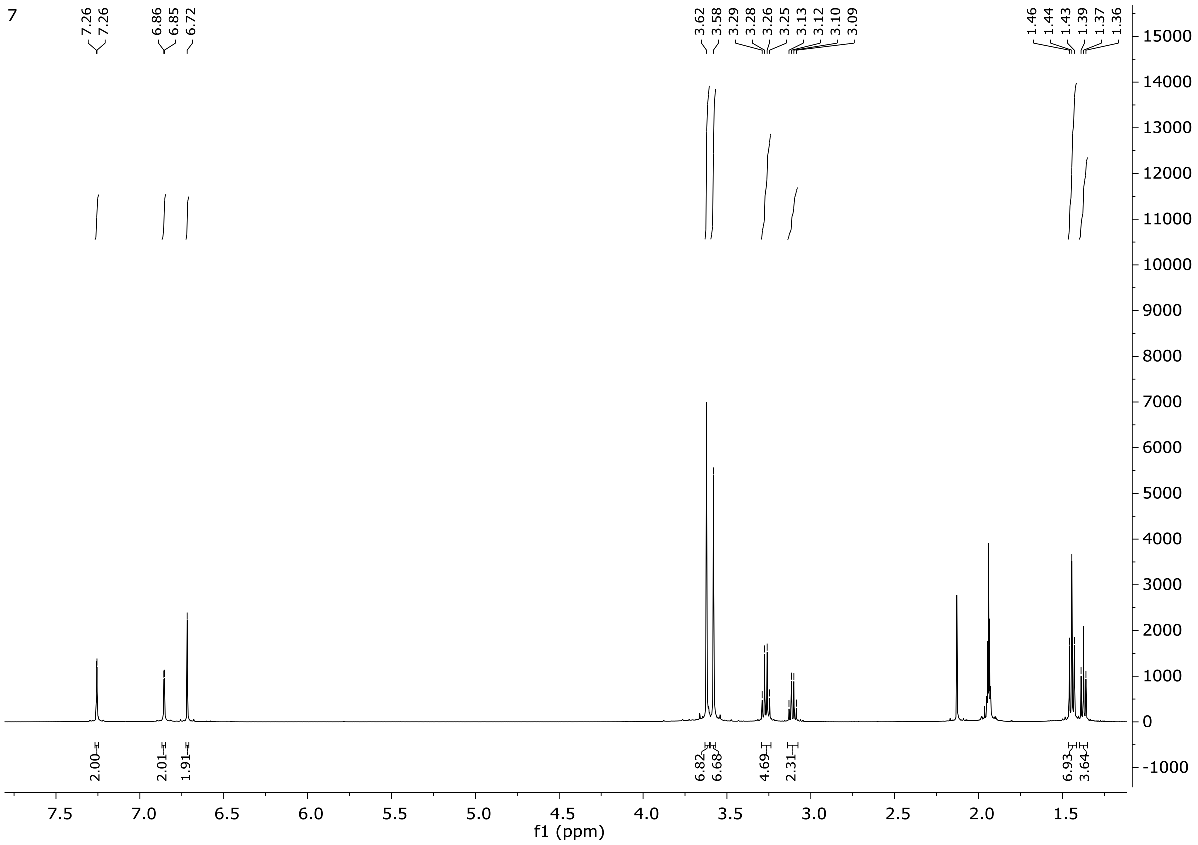

^{1}H NMR (500 MHz) spectrum of compound **7** recorded in CD_3CN.

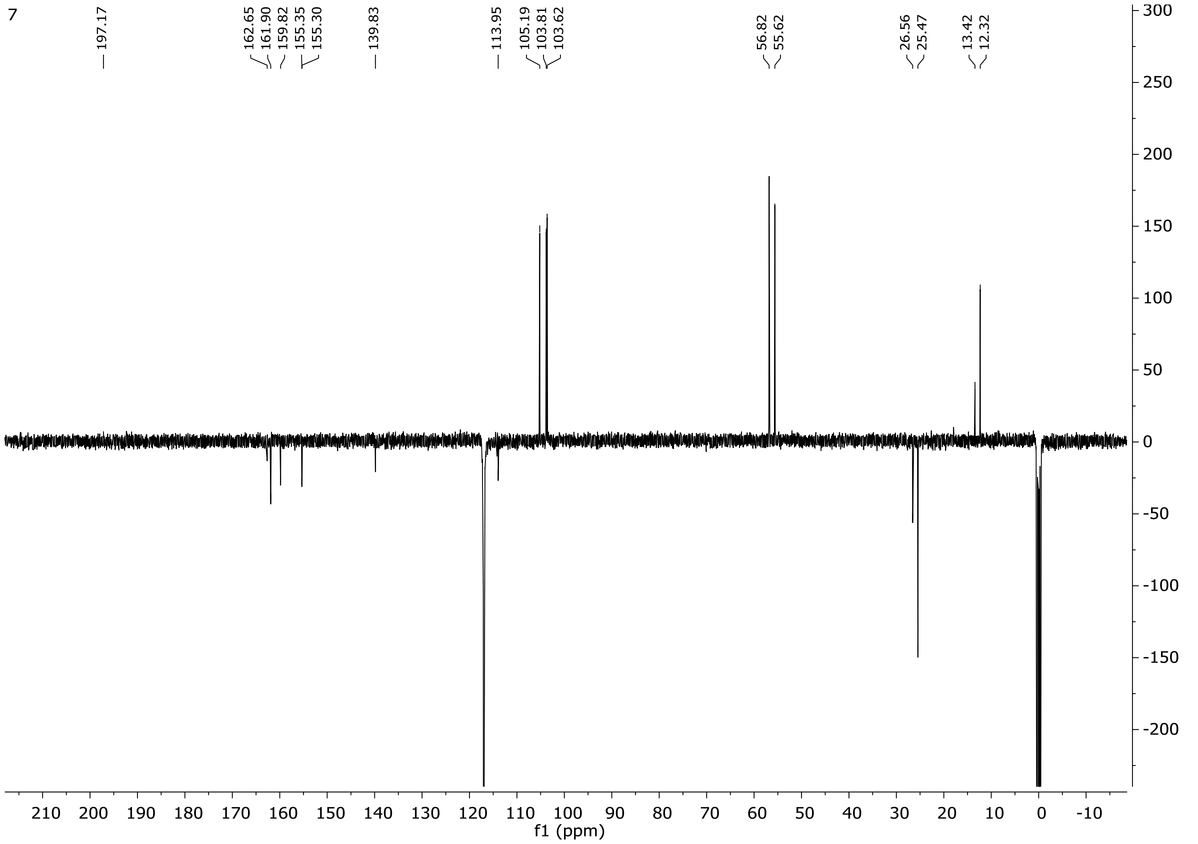

^{13}C APT NMR (126 MHz) spectrum of compound **7** recorded in CD_3CN.

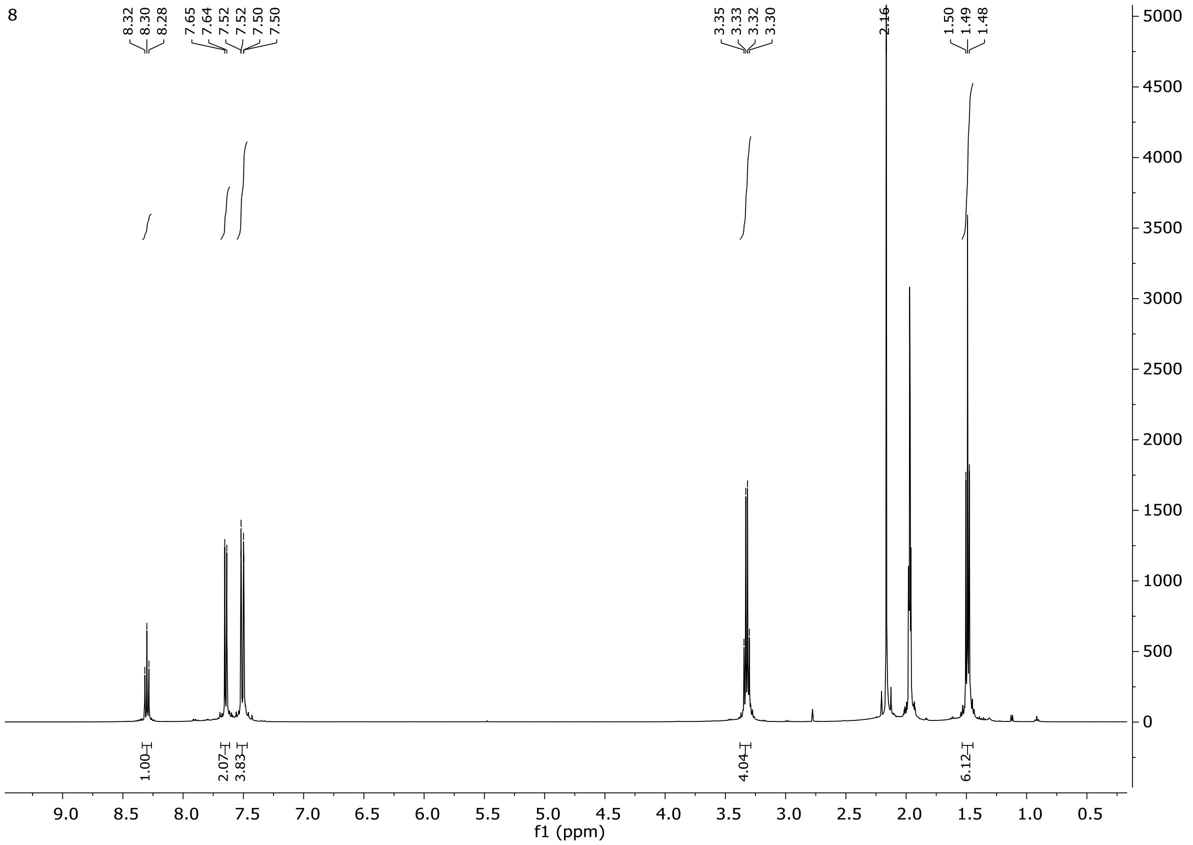

^{1}H NMR (500 MHz) spectrum of compound **8** recorded in CD_3CN.

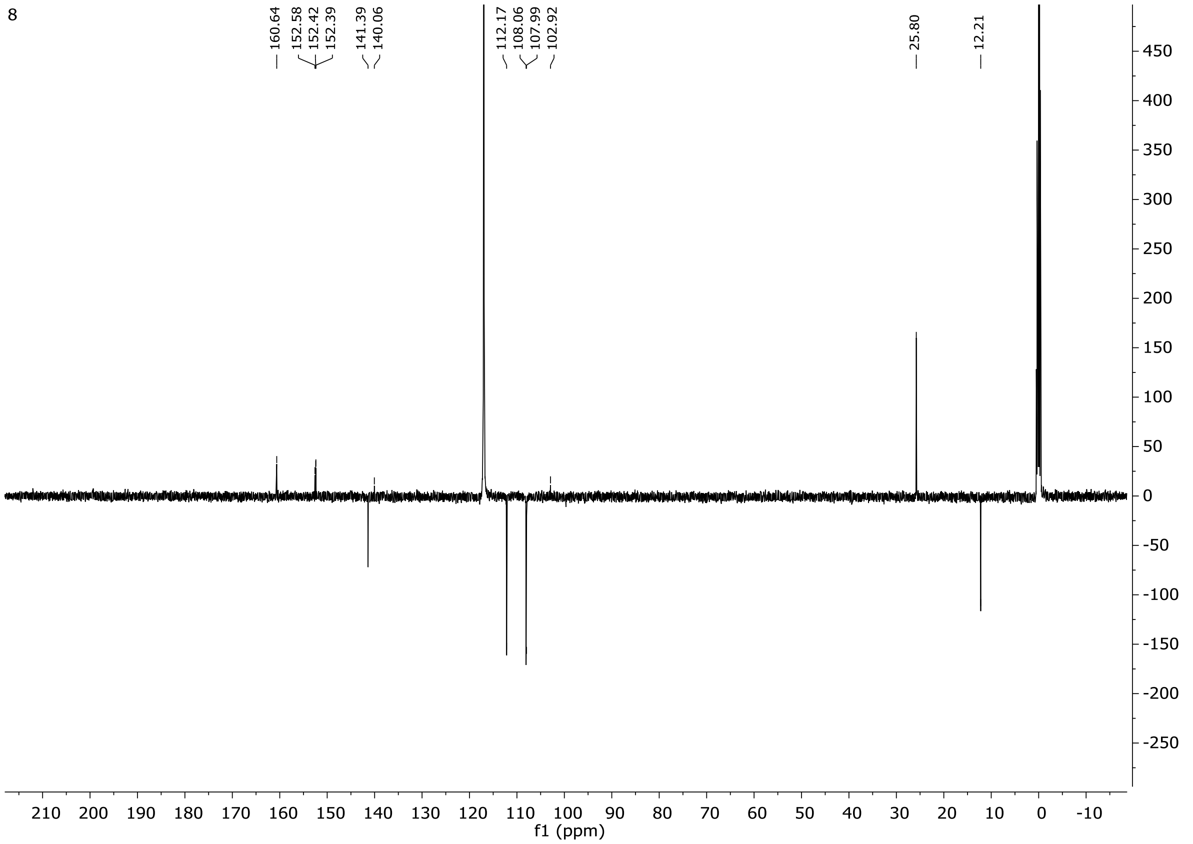

^{13}C APT NMR (126 MHz) spectrum of compound **8** recorded in CD_3CN.

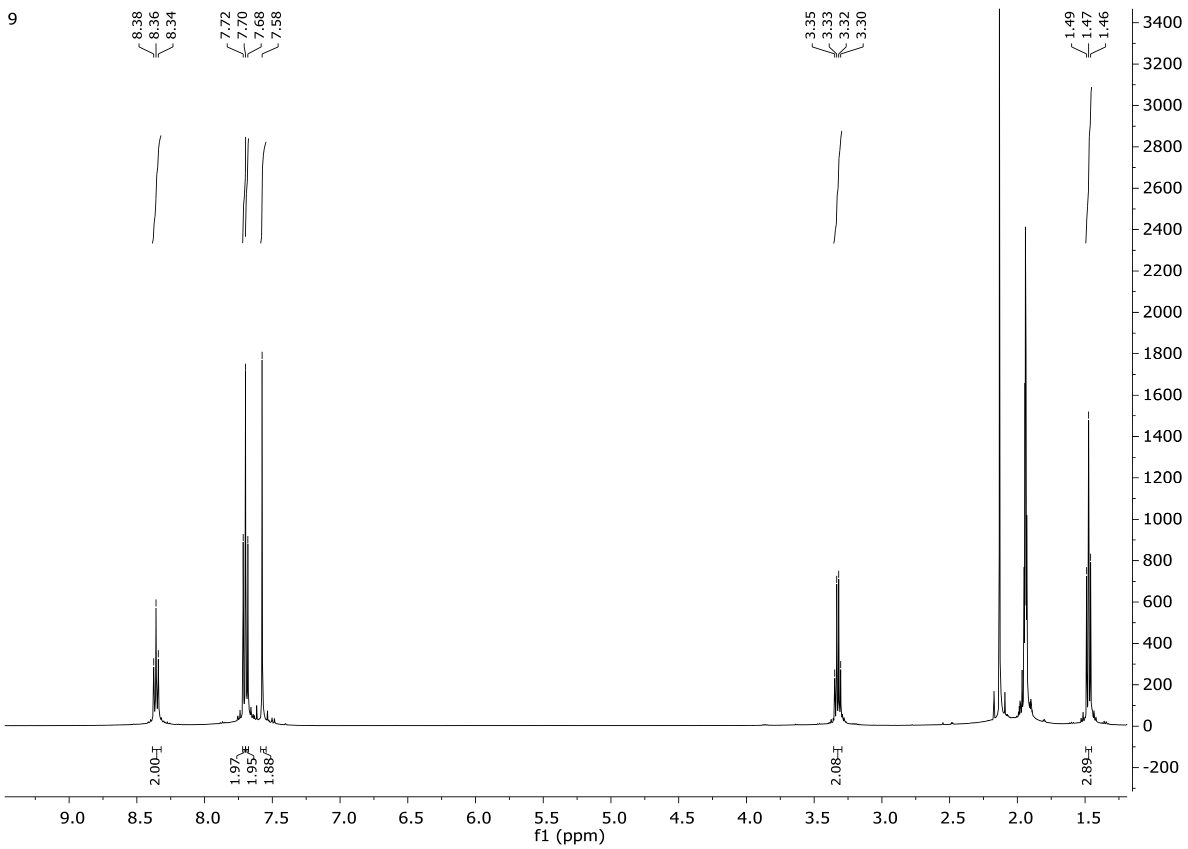

^{1}H NMR (500 MHz) spectrum of compound **8** recorded in CD_3CN.

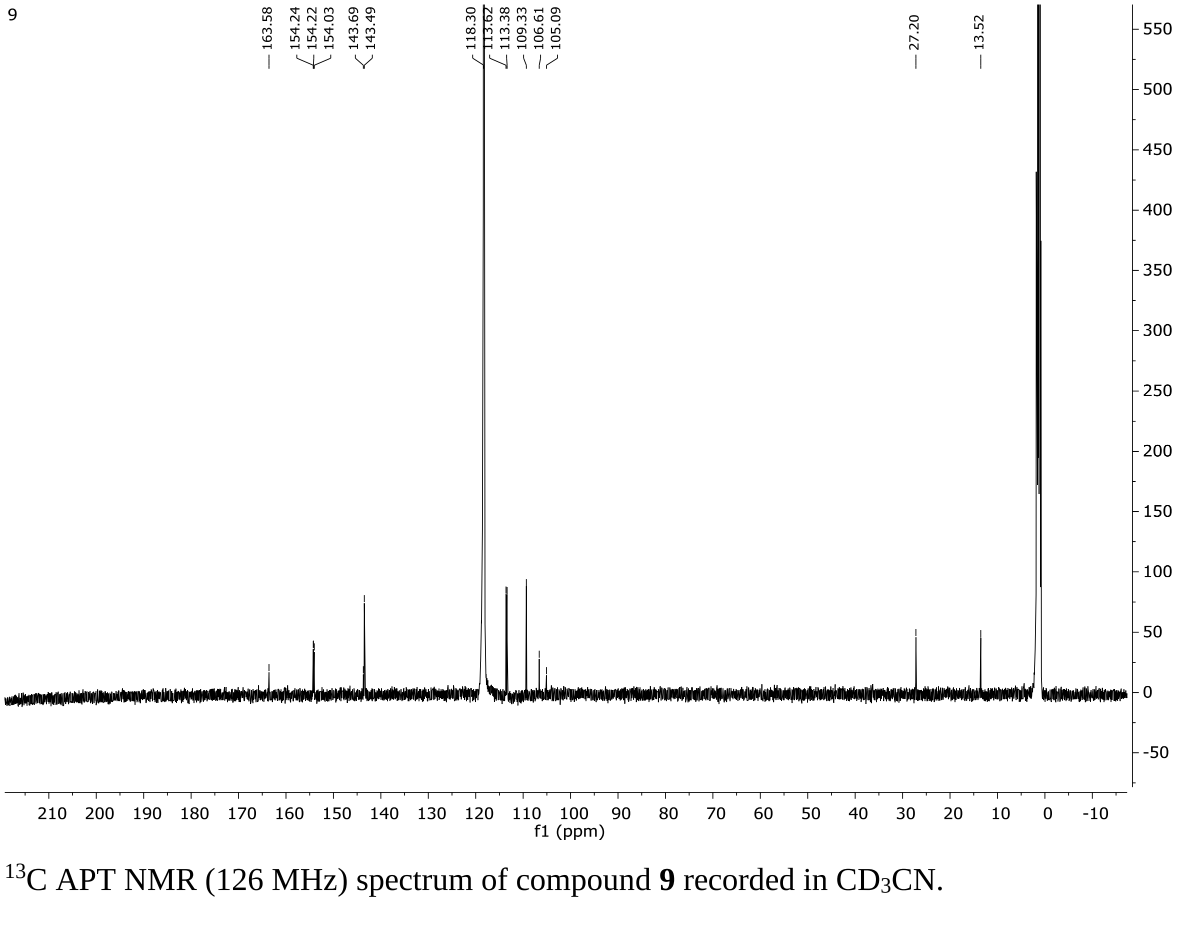

^{13}C APT NMR (126 MHz) spectrum of compound **9** recorded in CD_3CN.

Table S1: Absorption maxima and molar absorption coefficients (ε) of the different S_3 molecules in various solvents.

Compound	Solvent	λ_{max} (nm)	$\varepsilon_{\lambda max}$ ($M^{-1}cm^{-1}$)
S_3-TOTA$^+$ **6**	CH_2Cl_2	478	91850
	MeCN	469	77150
	MeOH	470	54700
S_3-ADOTA$^+$ **5a**	CH_2Cl_2	442	76700
		507	35400
	MeCN	435	67500
		504	30100
S_3-ADOTA$^+$ **5b**	CH_2Cl_2	442	53700
		507	25300
	MeCN	435	50600
		504	23900
S_3-acridinium **4a**	CH_2Cl_2	463	50000
	MeCN	457	47100
	MeOH	458	47500
S_3-acridinium **4b**	CH_2Cl_2	462	47700
	MeCN	457	46000
	MeOH	458	46700
S_3-xanthenium **7**	CH_2Cl_2	525	69900
	MeCN	520	60000
	MeOH	521	56200

References

1. Laursen, B. W.; Krebs, F. C.; Nielsen, M. F.; Bechgaard, K.; Christensen, J. B.; Harrit, N. *J. Am. Chem. Soc.* **1998**, *120* (47), 12255-12263, Article; Sørensen, T. J.; Laursen, B. W. *The Journal of Organic Chemistry* **2010**, *75* (18), 6182-6190.
